# Elevated Salinity Rapidly Confers Cross-Tolerance to High Temperature in a Splash-Pool Copepod

**DOI:** 10.1093/iob/obac037

**Published:** 2022-08-06

**Authors:** Mark W Denny, W Wesley Dowd

**Affiliations:** Hopkins Marine Station of Stanford University, 120 Ocean View Boulevard, Pacific Grove, CA 93950, USA; School of Biological Sciences, Washington State University, 100 Dairy Road, Eastlick G81, Pullman, WA99164, USA

## Abstract

Accurate forecasting of organismal responses to climate change requires a deep mechanistic understanding of how physiology responds to present-day variation in the physical environment. However, the road to physiological enlightenment is fraught with complications: predictable environmental fluctuations of any single factor are often accompanied by substantial stochastic variation and rare extreme events, and several factors may interact to affect physiology. Lacking sufficient knowledge of temporal patterns of co-variation in multiple environmental stressors, biologists struggle to design and implement realistic and relevant laboratory experiments. In this study, we directly address these issues, using measurements of the thermal tolerance of freshly collected animals and long-term field records of environmental conditions to explore how the splash-pool copepod *Tigriopus californicus* adjusts its physiology as its environment changes. Salinity and daily maximum temperature—two dominant environmental stressors experienced by *T. californicus*—are extraordinarily variable and unpredictable more than 2–3 days in advance. However, they substantially co-vary such that when temperature is high salinity is also likely to be high. Copepods appear to take advantage of this correlation: median lethal temperature of field-collected copepods increases by 7.5°C over a roughly 120 parts-per-thousand range of ambient salinity. Complementary laboratory experiments show that exposure to a single sublethal thermal event or to an abrupt shift in salinity also elicits rapid augmentation of heat tolerance via physiological plasticity, although the effect of salinity dwarfs that of temperature. These results suggest that *T. californicus*’s physiology keeps pace with the rapid, unpredictable fluctuations of its hypervariable physical environment by responding to the cues provided by recent sublethal stress and, more importantly, by leveraging the mechanistic cross-talk between responses to salinity and heat stress.

## Introduction

Organisms are subjected to daily, seasonal, and long-term fluctuations in multiple aspects of their physical environment. To survive, forage, compete, and reproduce, an individual's physiology must be able to respond appropriately to all combinations of these potential stressors and at all relevant temporal scales. In light of the rapid rate at which climate change is affecting the amplitude and trajectory of variation experienced by individual organisms (e.g., Harris et al. 2018), there is an urgent need to better understand the mechanisms by which organisms respond to their physical environment, and to identify the critical temporal scales over which these responses play out.

There are several challenges to developing this understanding; we highlight two. First, environmental variation is often unpredictable, particularly when rare, extreme events are considered (e.g., Jentsch et al. 2007; Denny et al. 2009; Smith 2011; Dowd et al. 2015). The consequences of environmental variation depend not only on the shape of the overall distribution of environmental states, but also on past experience and the timing and intensity of extremes (Dillon et al. 2007; Hoffmann 2010; Marshall and Sinclair 2010; Dowd and Denny 2020). These aspects render many traditional laboratory experimental designs inadequate for understanding natural variation. Second, the response to variation in one environmental factor may depend on patterns of variation of other stressors (e.g., Crain et al. 2008; Todgham and Stillman 2013; Jackson et al. 2021). The consequences of interactions between multiple fluctuating environmental variables are often complex: they may be compensatory (increasing, or at least maintaining, performance) or pathological (decreasing performance); in either case likely involving reallocation of resources to an environmental stress response (Kültz 2005). Regardless of the direction of performance change, the magnitude of the performance shift can be realized in additive, antagonistic (i.e., offsetting), or synergistic fashion (Folt et al. 1999; Gunderson and Stillman 2015; Paganini et al. 2014).

In the quest to overcome these challenges, the splash-pool copepod *Tigriopus californicus* can serve as a model organism. Confined to supratidal pools, these animals cannot escape the vagaries of their hypervariable environment, including extraordinary daily and seasonal fluctuations in temperature and salinity. Spurred on by the species’ ability to survive in such a stressful habitat, biologists have explored *T. californicus*’s thermal and salinity tolerances (*thermal tolerance*: Willett 2010; Kelly et al. 2012; Schoville et al. 2012; Pereira et al. 2013; Tangwancharoen and Burton 2014; Foley et al. 2019; Harada et al. 2019; Healy et al. 2019; Scheffler et al. 2019; Harada and Burton 2019, 2020; Tangwancharoen et al. 2020; Dinh et al. 2020; *salinity tolerance*: Burton and Feldman 1982, 1983; Goolish and Burton 1988, Burton 1991; Willett and Burton 2002; Kelly et al. 2016; DeBiasse et al. 2018; Foley et al. 2019; Lee et al. 2020). Complementing these physiological studies, population geneticists have described genetic differentiation among populations at both local and latitudinal scales (e.g., Burton et al. 1979, Burton and Feldman 1981; Burton and Lee 1994; Dybdahl 1994; Burton 1998; Kelly et al. 2012; Schoville et al. 2012; Harada et al. 2019; Tangwancharoen et al., 2020), and selection experiments have explored the potential for *T. californicus* to respond to changes in temperature and salinity through rapid evolution (e.g., Kelly et al. 2012, 2013, 2016, 2017; Griffith et al. 2020). Several efforts have been made to draw connections between the biochemical/physiological and the ecological/evolutionary levels of study. For example, a latitudinal gradient in heat tolerance and its underlying mechanism(s) is mirrored by a corresponding gradient in pool temperatures (Burton et al, 1979; Burton and Feldman 1981; Kelly et al. 2012; Schoville et al. 2012; Leong et al. 2018; Harada et al. 2019; Healy et al. 2019). By contrast, it is unclear whether there is a latitudinal gradient in salinity or salinity tolerance (Leong et al. 2018; Lee et al., 2020).

With the admirable goal of explaining *T. californicus*’s ecology and evolution through a mechanistic understanding of the species’ physiological interaction with environmental stressors, these efforts have generated numerous insights. In our opinion, this reductionist approach is the most promising path to predicting *T. californicus*’s response to future climate change, and the best way these copepods can serve as a model for predicting other species’ fates. However, difficulty arises when attempting to interpret our current understanding of *T. californicus*’s physiology and genetics in the context of the complexly variable conditions these copepods encounter in the field. The problem is fourfold.

First, our current understanding of the biochemical and physiological basis of *T. californicus*’s environmental tolerance is founded on experiments conducted in the laboratory, where animals are typically held for multiple generations at constant temperature and salinity prior to testing. Following this period of acclimation, animals are subjected to a shift in temperature or salinity: for salinity, an abrupt jump to a new level; for temperature, a ramp-up/ramp-down protocol (e.g., Willett 2010: Kelly et al. 2012, 2017; Schoville et al. 2012; Tangwancharoen and Burton 2014; Harada and Burton 2019). These experimental protocols have a distinct advantage in that they allow for definitive identification of physiological responses to a single-factor environmental shift. Acclimation to stable lab conditions also levels the playing field for population comparisons (e.g., Willett 2010, Kelly et al. 2016, 2017). However, because these experiments drastically simplify the environmental history animals experience before a shift in conditions, it is difficult to know how to translate their results to the complex variation encountered in the field.

Second, even within the simplified laboratory environment, experiments have (with few exceptions) examined *T. californicus*’s response only to a single stressful event. For instance, animals are subjected to a high temperature (either acute or chronic), and the subsequent survivorship, competitive ability, and physiological adjustments (one form of physiological plasticity) are measured (e.g., Willett 2010; Schoville et al. 2012; Tangwancharoen and Burton 2014; Kelly et al. 2016; Harada and Burton 2019; Harada et al. 2019). In nature, however, one stressful event is often swiftly followed by another (Denny et al. 2009; Dowd et al. 2015), and single-event experiments provide no information as to carryover effects: that is, whether an initial sublethal stress strengthens or weakens an animal's defenses to follow-on events (Williams et al. 2016). One exception (Kelly et al. 2012) used daily excursions from 19°C to 28°C and back as a “chronic” high-temperature regime. Animals raised with repeated stressful episodes acquired elevated heat tolerance compared to animals raised at constant 19°C, but it is unclear how many episodes were required to induce that elevated tolerance. In another exception, Kelly et al. (2017) showed that inter-population hybrids exposed to a single sublethal heat shock (34°C) increased tolerance to a more severe heat stress imposed a day later, but it is unclear whether this capacity exists in natural populations or how long it persists.

Third, although in nature temperature and salinity vary simultaneously, laboratory experiments typically hold one of these variables constant while measuring the effect of variation in the other. Exceptions are rare. Vittor (1971) examined the effect of three combinations of constant salinity and temperature on the rate of egg production in *T. californicus*: elevated salinity increased fecundity at low temperature (15°C) but decreased fecundity at higher temperatures (20 and 25°C). Edmands and Deimler (2004) examined the effects on fitness of different combinations of constant temperature and salinity. They noted significant variation among treatments; however, they did not report the nature of that variation. In the most instructive work to date, Kelly et al. (2016) maintained hybrid *T. californicus* populations at constant high and low salinities, observing that heat tolerance differed between lines selected over five generations: low heat tolerance in low salinity and (for males) higher tolerance in high salinity. Elevated thermal tolerance in animals selected for high salinity tolerance was—in response to heat shock—accompanied by up regulation of genes involved in protein stabilization. In short, substantial interaction between temperature and salinity is evident in *T. californicus*’s physiology. Although the nature and physiological basis for that interaction is beginning to emerge, much work remains to elucidate how copepods respond to the high-amplitude, rapid, co-occurring fluctuations in temperature, and salinity found in nature.

Lastly, we know surprisingly little about the actual distribution and temporal pattern of the environmental variables *T. californicus* encounters in the field. Correlations between genetic identity or thermal tolerance and environmental conditions are often drawn without actual measurements of pool temperature; instead, latitude is used as a proxy for temperature. This is problematic because there is substantial evidence that latitude is a poor predictor of maximum temperature for intertidal organisms (Helmuth and Hofmann 2001; Helmuth et al. 2002). Even when temperature measurements are available, they are often inadequate. Weekly or monthly snapshots of temperature and salinity (e.g., Egloff 1966; Vittor 1971; Powlik 1999) provide no information about the variation at higher frequencies that predominates in splash pools, and likely miss rare extreme events. While the advent of miniature data loggers has facilitated high-temporal-resolution measurements of tide pool temperatures (e.g., Leong et al. 2018; Harada and Burton 2019), these measurements have usually extended for only a few days to a few months. In the most extensive measurements to date, Kelly et al. (2012) measured temperatures hourly for two years in 19 pools in California and Oregon. They report descriptive statistics for the overall distribution of temperatures in their pools, but do not analyze the time course of temperature fluctuations. Critically, none of the high-resolution temperature data recorded to date are accompanied by simultaneous salinity measurements. Without a more thorough understanding of how temperature and salinity co-vary, it is difficult (if not impossible) to appropriately characterize *T. californicus*’s *in situ* physiology, compromising its utility as a model species.

In light of these difficulties, our goal in this combined field and laboratory study is threefold: (1) to document in detail the long-term co-variation in temperature and salinity in a representative splash pool, (2) to measure how the heat tolerance of the pool's *Tigriopus* population responds to the natural variation in these two stressors, and (3), informed by the predominant intervals between high-temperature events, to explore how a sublethal shift in either stressor affects subsequent heat tolerance. Our results suggest that *T. californicus* takes advantage of both the cross-talk between the responses to salinity and thermal stress and the cues provided by sublethal stress, allowing its physiology to keep pace with the rapid, unpredictable fluctuations of its hypervariable physical environment.

## Methods

### Site

The experimental splash pool is located near the peak of a rocky outcrop on the shore at Stanford's Hopkins Marine Station (HMS), Pacific Grove, California, USA (Google Earth coordinates: 36.621800 N, 121.905073 W). The pool's altitude was measured using a total station (211D, Topcon Inc., Tokyo, Japan) with reference to a USCGS benchmark approximately 100 m away. Its volume was ascertained by emptying it with a large syringe and measuring the extracted water with a volumetric cylinder. Water was then added back to the pool in measured amounts, and photographs were taken from above (with a ruler in the picture for scale) to measure the pool's surface area as a function of its volume.

The lowest point in the experimental pool is 5.11 m above mean lower low water (MLLW), and the maximum depth of the pool is 0.11 m. Maximum pool capacity is 8.77 liters, and pool surface area increases approximately linearly with pool volume (Fig. S1). The rocky peak on which the pool sits is separated from the mainland by a “pass” 3.11 m above MLLW, ensuring that freshwater runoff from the mainland cannot enter the pool. Thus, the only input of water to the pool is from wave splash and any rain collected by the small catchment basin of the surrounding rocks.

During an unusual period of calm seas in September, 2021, the pool evaporated to near dryness. To prevent the probable loss of the pool's *Tigriopus* population during this episode, seawater collected adjacent to the site was twice added to the pool, mimicking the effect of wave splash. Otherwise, pool volume was not manipulated.

### Environmental variables

Water temperature in the pool (which for these tiny animals equals body temperature; [Denny 1993, 2016]) was measured using iButton data loggers (Maxim Integrated, San Jose, California, USA) secured in the middle of the water column by a small, perforated plastic pipe fitting that screwed into a coupling glued to the rock. The loggers recorded temperature every 20 min with a resolution of 0.5°C. Recording was initiated in October 2019, and continued through January 2022, interrupted by a COVID-19-related hiatus from April to July of 2020. A total of 624 days of pool temperatures were recorded.

Pool salinity was measured to the nearest part per thousand (ppt) with a handheld refractometer, the accuracy of which was confirmed using laboratory-mixed solutions of NaCl. Samples for measurement were drawn from the middle of the water column adjacent to the temperature loggers. Salinity measurements were initially taken only in conjunction with the collection of copepods for heat-tolerance measurements (see below), but from August 2021 through January 2022 measurements were made daily, resulting in a total of 163 consecutive observations. On days when salinity was >100 ppt (the maximum value on the refractometer scale), pool water was diluted in known proportion with deionized water before measurement.

### Heat-tolerance indices

Copepods’ tolerance of elevated temperature was assessed by measuring two standard indices (e.g., Kelly et al. 2012, 2016; Harada et al. 2019; Griffiths et al. 2020). (1) *LT*_50_, the temperature required to kill half the individuals tested, directly assesses potential mortality from elevated temperatures. (2) Knockdown temperature, the temperature at which individuals abruptly cease swimming, is a convenient indicator of disruption to homeostasis; it potentially equates to “ecological death” (a nonmotile copepod cannot eat or reproduce), but it is a less direct measure of population consequences than is *LT*_50_.

*LT*_50_: *LT*_50_ was measured using a programmable gradient thermal cycler (MasterCycler Gradient, model 5331, Eppendorf Inc., Enfield, Connecticut, USA). *Tigriopus* were collected from the experimental pool using a small sieve; care was taken to collect animals from both the substratum and the entire water column to ensure representation from the whole population. Animals were returned to the laboratory, and immediately transferred to transparent 0.2 mL PCR tubes. For each tube, several individuals (mean = 4.5) were sucked haphazardly from the field collection using a transfer pipette, and expelled along with 0.1 mL of the pool water into the tube. Because these measurements were intended to reflect the effect of temperature on the population as a whole, no effort was made to separately sample males or females, or individuals of particular developmental state. Tubes were then sealed and inserted into the thermal cycler. The salinity of the pool water was measured to the nearest ppt using the handheld refractometer.

**Fig. 1:**
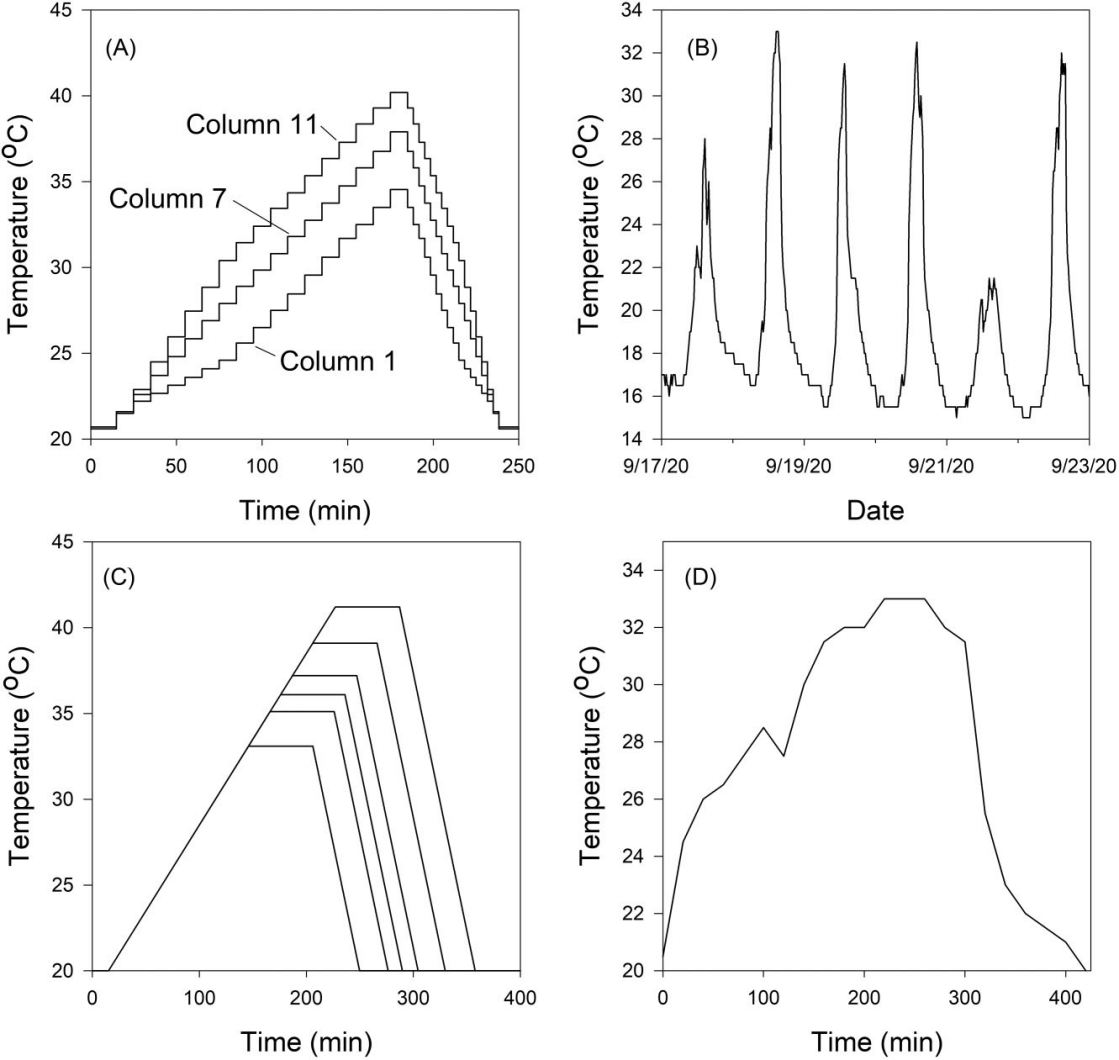
The experimental temperature ramps mimic thermal history in the field. (**A**) Thermal ramps used to measure *LT*_50_ of field-fresh*T. californicus*. (**B**) Representative thermal history in the experimental pool. (**C**) Thermal ramps used to measure *LT*_50_ of copepods subjected to an abrupt shift in salinity. (**D**) Representative thermal variation in a single day (September 18, 2020) in the experimental pool.

A heat ramp was then applied to the experimental individuals (Fig. 1A). From the initial temperature of 20°C, temperature was gradually increased to a peak temperature and then more rapidly decreased back to 20°C, a time course mimicking the pattern of temperature variation in the field (Fig. 1B, D) and similar to that used by Harada and Burton (2019, 2020) and Dinh et al. (2020). The peak temperature imposed on a given tube varied with the tube's location in the cycler's plate, with peak temperature increasing from lowest (34.6°C) in column 1 to highest (40.2°C) in column 12. Thus, in these trials the ramping rate varied slightly among peak-temperature groups (5.5 to 7.6°C hr^–1^ for the ramp up, −16.4 to −22.7°C hr^–1^ for the ramp down), but the total duration of exposure to elevated temperature was held constant. Five replicate tubes were loaded into each column. Temperatures in each column were calibrated using 22-gauge thermocouples inserted into 0.1 mL of water in 0.2 mL PCR tubes, monitored with a thermocouple thermometer (Model HH23, Omega Engineering, Norwalk, Connecticut, USA). Thermocouples were calibrated at water temperatures varying from 20°C to 42°C, in turn measured to the nearest 0.1°C using a research-grade mercury thermometer.

Survival subsequent to heat shock was assessed by examining each tube two days later. Individuals (adults and late-stage copepodids) were viewed in their tubes using a dissecting microscope. They were scored as alive if they were actively moving, dead if they were inactive. Summing the results across the five tubes in each column provided an estimate of the fraction of individuals surviving that column's maximum temperature. *LT*_50_ was estimated by logistic regression of these survival-vs.-peak-temperature data in *R* (Ver. 4.1.1, The *R* Foundation for Statistical Computing) using the *glm* function with a binomial link. Air in the 0.1 mL headspace in each tube was refreshed daily by opening the tube, spritzing the headspace with 8 mL of room air, and resealing the tube.

*Knockdown Temperature*: Copepods were collected from the experimental pool as described above and returned to the laboratory, where 20–30 individuals were immediately transferred to each of two 3 mL glass test tubes along with the pool water in which they were collected. The tubes were placed in a water bath at 20°C. The salinity of the pool water was measured with the handheld refractometer.

A 22-gauge thermocouple in each tube measured the water temperature, which was displayed twice per second on a small LCD screen held behind a narrow, see-through tank of water maintained at 42°C by a circulating water bath. To initiate an experiment, one tube (with its copepods) was taken from the 20°C water bath and inserted into the high-temperature tank alongside the thermocouple display so that copepods’ activity could be video recorded (iPhone X, Apple Corp., Cupertino, California, USA) simultaneously with the water temperature in the tube. As temperature increased from its initial 20°C (at approximately 10°C min^–1^), activity among the test animals increased over the course of approximately 1.5 to 2 min until, at a certain temperature individuals ceased swimming and drifted to the bottom of the tube. The temperature at which the last individual reached bottom was recorded as the knockdown temperature. The tube was immediately returned to the 20°C bath, where its temperature rapidly decreased. While the first tube was returning to 20°C, the experiment was repeated with the second tube. Tubes were alternated in this fashion (the knockdown temperature in each tube being measured approximately every 4 min) until five measurements of knockdown temperature were recorded for each. Knockdown temperatures were consistent across these repeated trials for a given group of copepods; no trend either up or down was noted, so the average of the five trials for each tube was used in subsequent analyses.

Variation of the measured *LT*_50_ and knockdown temperatures of field-collected animals was estimated as a function of the environment using a generalized additive linear model:

(1) \begin{equation*}Y = aS + b{S}^2 + cM + dT\end{equation*}

Here, *Y* is the variable being modeled (*LT*_50_ or knockdown temperature), *S* is salinity (ppt) at the time of collection ( = salinity during the test), *M* is an index of season (*M* = cos[*2π D*/*N*] where *D* is the year day of the day on which animals were collected and *N* is the number of days in a year [365.25]), and *T* is the maximum pool temperature on the day before experimental animals were collected. Calculations were conducted using the function *lm* in *R* (Ver. 4.1.1, The *R* Foundation for Statistical Computing).

### Laboratory study: Effects on heat tolerance of salinity acclimation and acute salinity change

To explore potential interactive effects of chronic and acute salinity changes on heat tolerance, we acclimated adult copepods to seawater of either 30 or 60 ppt (temperature 22°C) for 4–11 days. Animals were maintained in ∼500 mL jars and fed once per week with a mixture of *Spirulina* and fish flakes. All animals were collected in July and August 2015 from a single splash pool at Hopkins Marine Station, on the same rock outcrop as the pool from which field monitoring data were collected. Immediately before assaying heat tolerance, copepods from each acclimation group were transferred to one of five test salinities: 20, 35, 60, 80, or 100 ppt.

Heat tolerance in this experiment was assayed in a thermocycler (PTC-100, MJ Research Inc., Waltham, Massachusetts) by exposing groups of animals (*N* = 9–20 per treatment group, with 4–5 adults in each 0.6 mL microcentrifuge tube; total *n* = 660) to a ramping protocol that began at 22°C immediately after animals were introduced to the new salinity, and reached one of a range of peak temperatures (33.1, 35.1, 36.1, 37.2, 39.1, or 41.2°C). No mortality was noted with transfer to a new salinity, and all animals were swimming at the initiation of the heat ramp. For these assays the temperature ramping rate was held constant at 0.1°C min^–1^ (6°C h^–1^), very close to the average maximum rate measured in our experimental pool (5.3°C h^–1^). Once the peak temperature was reached, the animals were held at that temperature for 1 h before ramping the temperature back down to 20°C at −0.3°C min^–1^ (−18°C h^–1^) (Fig. 1C). One peak temperature could be assayed per day for all acclimation-by-acute-salinity combinations; the temperature order was haphazardly scrambled to avoid systematic biases. Following the temperature ramp, animals were maintained at room temperature (∼22°C), and survival was scored as the number of individuals swimming 4 days after the temperature ramp. The median lethal temperature (*LT*_50_) was estimated from a logistic regression as with the measurement of field *LT*_50_ (see above).

### Laboratory study: Carryover effects of a sublethal heat stress on heat tolerance

To augment our measurements of thermal tolerance in field-acclimatized copepods, we conducted laboratory experiments to probe the carryover effects of a single sublethal heat exposure. Copepods were collected from the experimental pool and maintained at room temperature (mean = 20.1°C, s.d. = 1.4°C) and a salinity of 38–40 ppt in the laboratory for at least 6 days (average 19.8 days) to minimize any effects of thermal and salinity variation experienced in the field. These lab populations were held in 500 mL of seawater and fed finely ground food (a combination of *Spirulina* and fish flakes) 1–2 times per week. Individuals were transferred to 0.2 mL PCR tubes and inserted into the Eppendorf thermal cycler as described above. All tubes were then subjected to a cycle of elevated temperature similar to that of Fig. 1A, but peaking at 35°C for all samples. This peak temperature was chosen to provide a substantial but sublethal heat shock; it is near the maximum temperature recorded in the experimental pool and has been shown to induce production of heat-shock proteins in *T. californicus* (Harada and Burton 2020). The vast majority (98%) of animals survived this initial exposure. The *LT*_50_ of individuals surviving the initial shock was then measured (as described above for freshly field-collected animals, see *Heat tolerance indices*) 1, 2, or 3 days after the shock. Air in the headspace of each tube was refreshed daily. As a control, a separate set of copepods from the same beaker was sampled and inserted into the thermal cycler, but these tubes were held at 20°C rather than receiving the initial heat ramp; their *LT*_50_ 1, 2, or 3 days later was measured as for the pre-stressed individuals. Each experiment (pre-stress and control; 1,2, and 3 day lags) was repeated 6–7 times, and the results were examined using control-vs.-experimental paired-sample *t*-tests to document any effects of pre-stress on heat tolerance.

### Time-series analyses

The time series of the splash pool's temperature and salinity were analyzed to characterize the distribution of each factor and the patterns of temporal variation and co-variation in these environmental stressors.

*Autocorrelation*: The autocorrelation function (ACF) provides information regarding the temporal scale at which a given environmental factor varies (Priestley 1981; Chatfield 1984). We calculated separate ACFs for daily maximum temperature and the corresponding salinity according to Eq. 2.2 of Chatfield (1984). For daily maximum temperature, separate ACFs were calculated for each data set retrieved from the iButton loggers (25 sets, each typically 28 days long), and the ACFs were averaged across sets to provide values for both the mean and 95% confidence limits of ACF estimates. When calculating the ACF for salinity, days affected by the artificial replenishment of the pool in conjunction with the near-extinction event of September 2021 (see above) were excised from the data; 95% confidence limits were calculated according to Chatfield (1984, pg. 25). As an index of the temporal scale of temperature and salinity variation, we use the decorrelation time, the temporal lag at which the ACF is first statistically indistinguishable from zero at the *p* = 0.05 level.

*Cross Correlation*: In contrast to the ACF, which describes the temporal pattern in which a single factor varies through time, the cross-correlation function (CCF) is an index of how variation in one environmental factor is temporally correlated with another (Priestley 1981; Chatfield 1984). We calculated the CCF for salinity and daily maximum temperature according to Eqs. 8.4 and 8.5 of Chatfield (1984), with 95% confidence limits calculated as for the salinity ACF.

*Inter-Event Intervals*: The interval *i* between each potentially stressful thermal event (daily maximum temperature ≥24.5 ^o^C [the top 5% of recorded temperatures] or 30°C [the top 0.5% or recorded temperatures]) and the next such event was recorded. The distribution of the *n* intervals was expressed as the estimated probability that a randomly chosen interval would be greater than a given length. To this end, the intervals were ranked in ascending order of length from *x* = 1 to *n*; ties being assigned their average rank. The estimated probability that the length of a random interval is $\ge $*i* is *P* = 1–[*x*/(*n* + 1)] (Coles 2001).

In addition to these primary metrics of temporal pattern, additional analyses were carried out to estimate (1) the conditional probability of encountering elevated temperatures and (2) the return time of extreme temperatures. Details of these ancillary analyses are given in the Supplement.

## Results

### Salinity variation

**Fig. 2:**
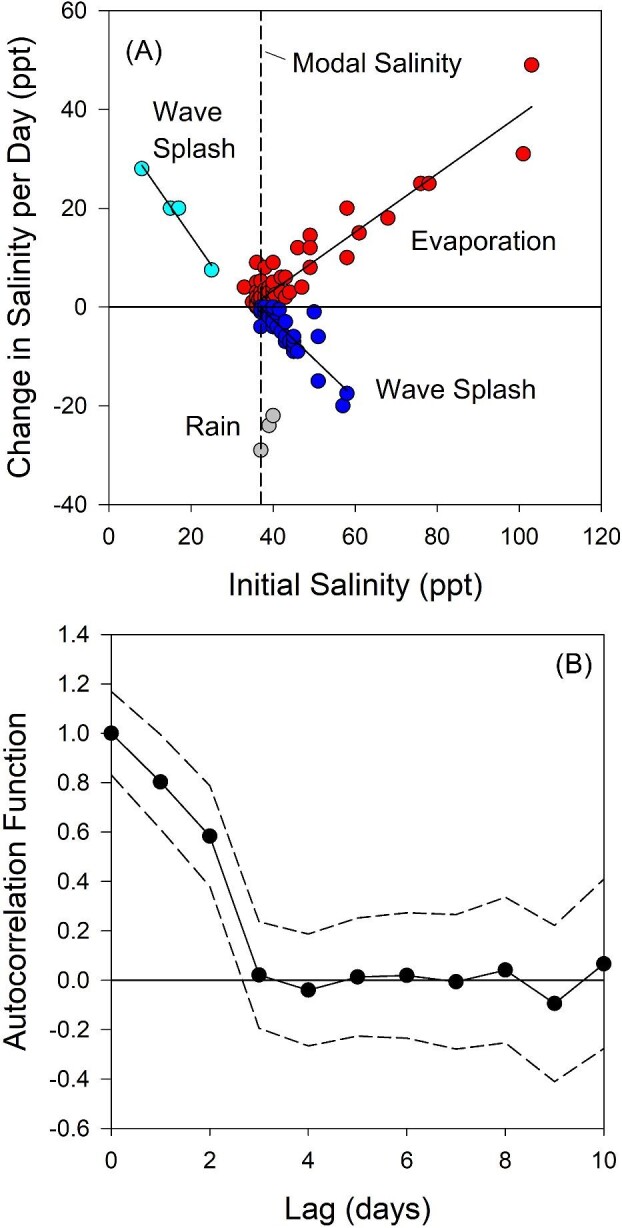
Patterns of salinity variation in the experimental pool. (**A**) Rate of change of salinity in various situations. When salinity is above the mode (37 ppt), it can increase as water evaporates (red dots) or decrease as seawater at ∼35 ppt is splashed into the pool (blue dots). In both cases, the rate of change depends on initial pool salinity. For evaporation, Δ*S* = 0.5901*S*_0_ + 20.224, where Δ*S* is the rate of change in salinity (ppt day^–1^) and *S*_0_ is the initial salinity (*r*^2^ = 0.877). For wave splash, Δ*S* = −0.797*S*_0_ + 29.331 (*r*^2^ = 0.799). When salinity is below the mode, it can increase at a rate that depends on initial salinity as seawater is splashed into the pool (cyan dots); Δ*S* = −1.1951*S*_0_ + 38.295 (*r*^2^ = 0.974). Salinity can decrease from the input of rainwater (0 ppt, grey dots). (**B**) Current pool salinity is significantly autocorrelated with future salinity for ∼2.5 days. Dashed lines are 95% confidence limits.

Measured salinities in the pool varied from 8 ppt to 190 ppt, with a mode of 37 ppt (Fig. S2A). Salinities above 60 or below 30 ppt (the acclimation salinities used in our salinity-shift experiments) are rare, accounting for 10% and 3% of the readings, respectively. For salinities initially above that of ambient seawater (∼35 ppt), the rate at which salinity increases by evaporation increases linearly as a function of current salinity (Fig. 2A), ranging up to 49 ppt day^–1^. This is a predictable consequence of the fact that the pool's surface area is proportional to its volume (see the Supplement: Rates of Change of Salinity). Elevated salinities can be instantaneously reduced by wave splash; the higher the initial salinity, the greater the decrease (Fig. 2A). By contrast, reduction in salinity by freshwater input occurs over several hours during rainstorms, and in our measurements was maximally −29 ppt day^–1^ (Fig. 2A). For salinities initially below that of ambient seawater, wave splash can increase salinity instantaneously (Fig. 2A).

In our overall salinity time series, elevated salinities tend to occur from April to October (Fig. S2B). The continuous September–January series is shown in Fig. S2C. The decorrelation time for salinity variation in this series is approximately 2.5 days (Fig. 2B). The pool's *Tigriopus* population was not noticeably affected by extremes in salinity; dense populations were evident the day after the pool returned to near-mean salinity from elevated values. Animals at both 8 and 190 ppt were not moving, but they began to swim actively within an hour after transfer to 38 ppt.

### Temperature variation

Daily maximum temperatures in the pool ranged from 11 to 36°C, with a mean of 21.2°C. The distribution was bimodal, with modes at 16°C and 26°C (Fig. S3A). Typically, temperature rose relatively slowly from sunrise until early afternoon, and then cooled more swiftly (Fig. 1B, D). On days with maximum temperature ≥25°C, the maximum rate of heating ranged from 3 to 10.5°C hr^–1^; the rate of heating was positively correlated with daily maximum temperature (Fig. S3B). The statistics of extremes suggest that the maximum temperature that will ever occur in the pool given current environmental variability is 36.4°C (see the Supplement: Additional Time-Series Analyses).

**Fig. 3:**
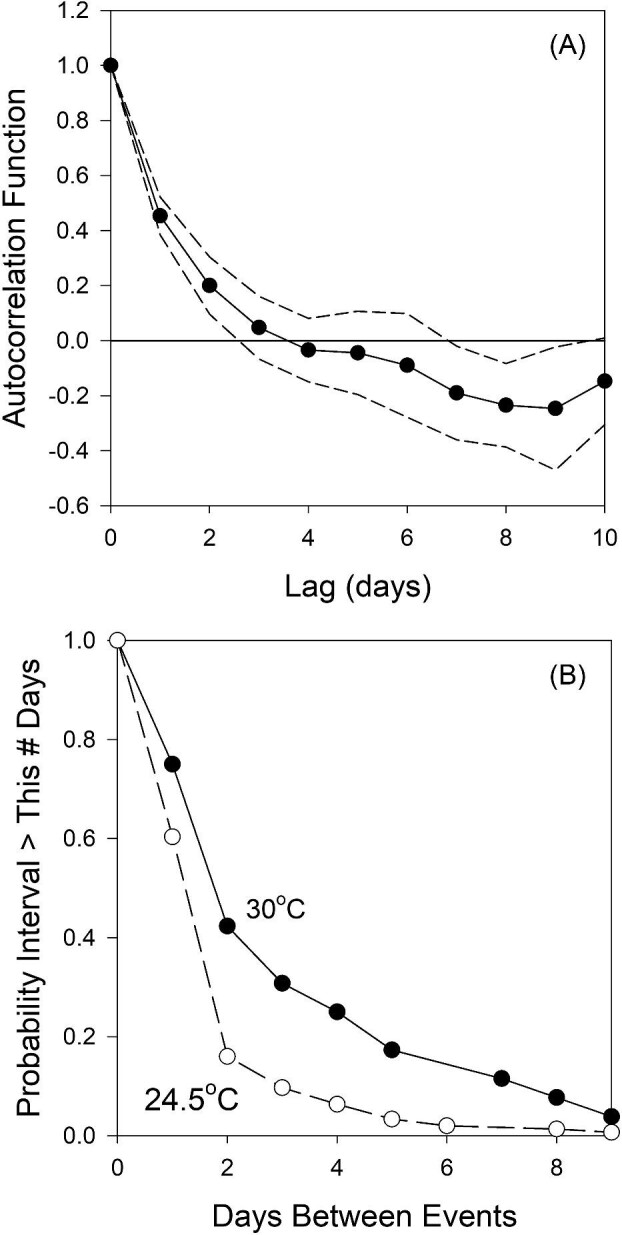
Patterns of temperature variation. (**A**) Current daily maximum temperature is autocorrelated with future maxima for only ∼2.5 days. (**B**) The probability that the interval leading to the next event is greater than a given duration decreases rapidly with inter-event interval. Here, events are defined by a daily maximum temperature ≥24.5°C (dashed line, open symbols) or 30°C (solid line, filled symbols).

The decorrelation time of daily maximum temperatures is approximately 2.5 days (Fig. 3A), similar to that of salinity. The probability that an inter-event interval is greater than a given length decreases rapidly with increased interval length (Fig. 3B). If an extreme event is defined as a daily maximum temperature ≥24.5°C, there is only a 10% probability that the next interval is greater than 3 days long. For extreme events ≥30°C, the chance that the next interval is greater than 3 days long is 30%. In sum, daily maximum (and minimum) temperatures have well-defined annual cycles, but, within a season, potentially stressful high temperatures are unpredictable more than 2–3 days in advance.

### Temperature-salinity cross-correlation

**Fig. 4:**
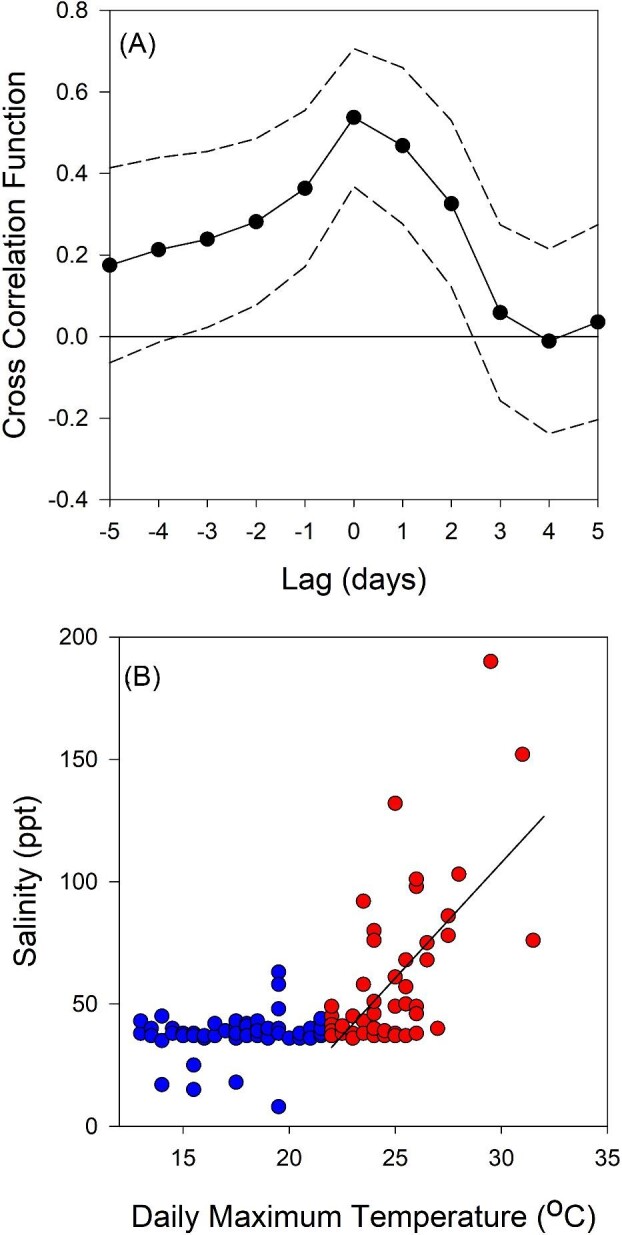
Salinity and daily maximum temperature are correlated. (**A**) The CCF between temperature and salinity; dashed lines are 95% confidence limits. (**B**) When daily maximum temperature is ≥22°C, salinity is (on average) elevated: *S* = 9.427*T*_max_ −175.06 (*r*^2^ = 0.423).

Overall, the pool's salinity is significantly correlated with maximum daily temperatures less than ∼3 days in the past, and up to ∼2.5 days in the future (Fig. 4A). Further inspection revealed that the correlation between salinity and temperature depends on the range of temperatures being examined. At temperatures <22°C there is no significant correlation between daily maximum temperature and salinity (*P* = 0.171). However, for daily maxima $\ge $22°C, there is a significant correlation (*P* ≪ 0.001) that explains 42% of the variance (Fig. 4B).

### Temporal variation in heat tolerance of freshly collected copepods

**Fig. 5:**
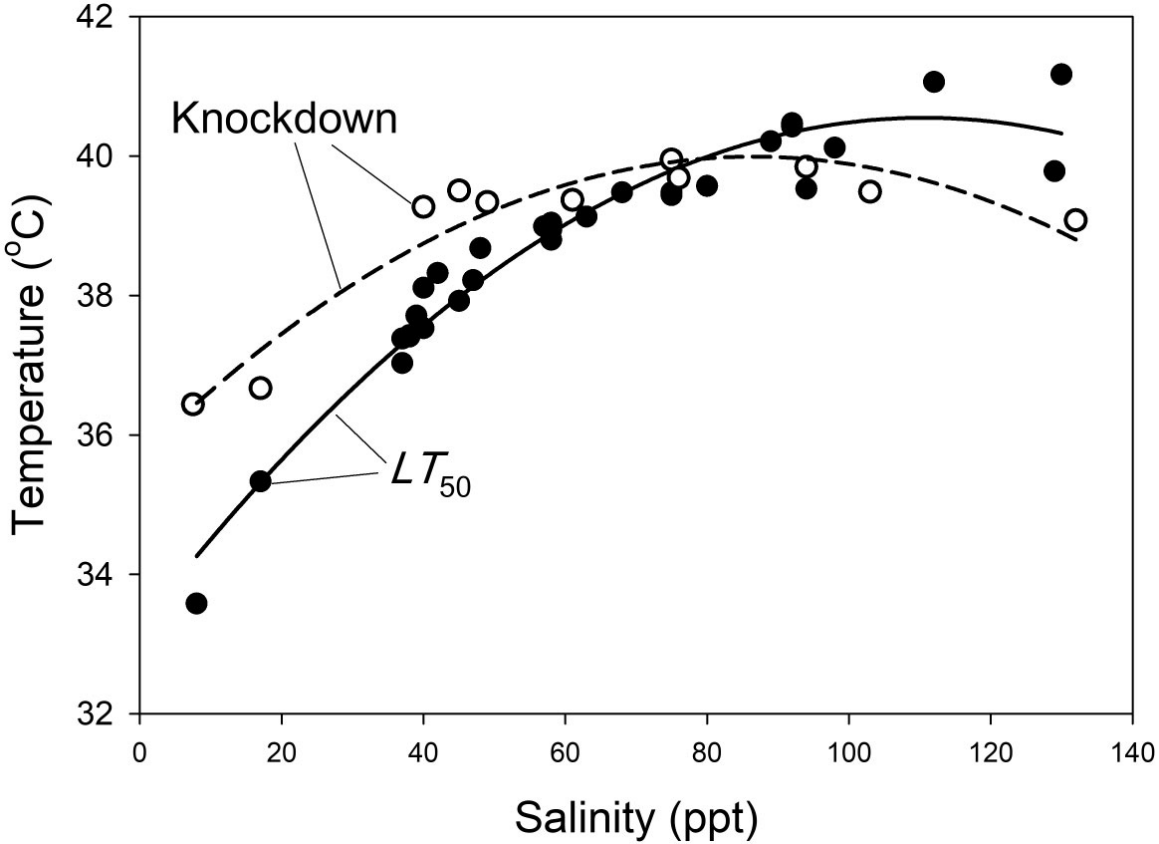
Thermal tolerance varies as a function of salinity. The *LT*_50_ (filled symbols) of field-fresh *T. californicus* increases with increasing pool salinity. By contrast, knockdown temperatures (open symbols) plateau above 40 ppt. The solid (*LT*_50_) and dashed (knockdown) lines are quadratic fits. *LT*_50_ = −5.977 × 10^–4^*S*^2^ + 0.1332*S* + 33.24 (*r*^2^ = 0.949); knockdown temperature = −5.740 × 10^–4^*S*^2^ + 0.0993*S* + 35.70 (*r*^2^ = 0.919).

**Fig. 6:**
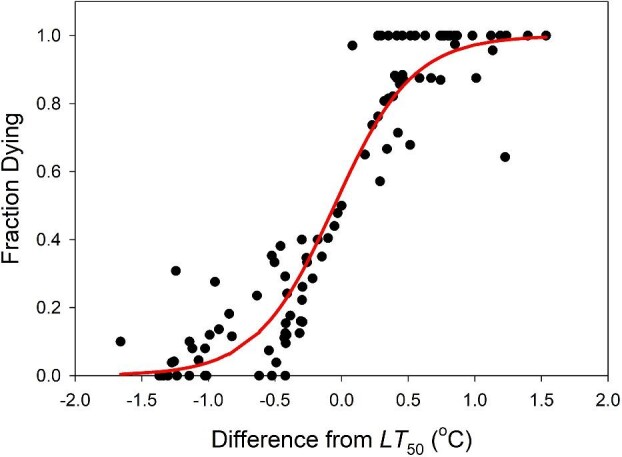
There is only a small difference in the imposed temperatures that results in 0% versus 100% of animals dying. The red line is the logistic fit to the data.

The *LT*_50_ of freshly-collected copepods increased with increasing salinity (Fig. 5), ranging from 33.7°C at 8 ppt to a peak of 41.2°C at 130 ppt. The general additive model (Equation 1) revealed that *LT*_50_ had no significant correlation with season or daily maximum temperature (*P* = 0.284, 0.835, respectively). After eliminating these non-significant factors from the model, the remaining second-order fit of *LT*_50_ to salinity accounts for 94.9% of the total variation in *LT*_50_. In these *LT*_50_ tests, the fraction of individuals dying increased from 0% to 100% over a narrow temperature range (Fig. 6), indicating that, if tolerance differs between sexes or among developmental stages, the variation is slight and much smaller than the overall range of measured *LT_50_* values.

As with *LT*_50_, knockdown temperature of freshly collected copepods increases with increasing salinity at salinities below 40 ppt, but it had no significant correlation with salinity at higher salinities (*P* = 0.889, Fig. 5). The range of knockdown temperatures was smaller than that of *LT*_50_ (36.4°C to 40.0°C). Knockdown temperature had no significant correlation with either season or maximum temperature (*P* = 0.202, 0.209, respectively). The remaining second-order fit of knockdown temperature to salinity accounts for 91.9% of the total variation in knockdown temperature.

### No effect of laboratory acclimation on baseline heat tolerance

The *LT*_50_ of animals maintained in the laboratory at 38–40 ppt for 6 to 46 days (mean *LT*_50_ = 37.96°C) did not significantly differ from animals freshly-collected at similar salinities in the field (37 to 42 ppt, mean = 37.94°C; *t*-test, *P* = 0.93).

### Response of LT_50_ to chronic and acute salinity change

**Fig. 7:**
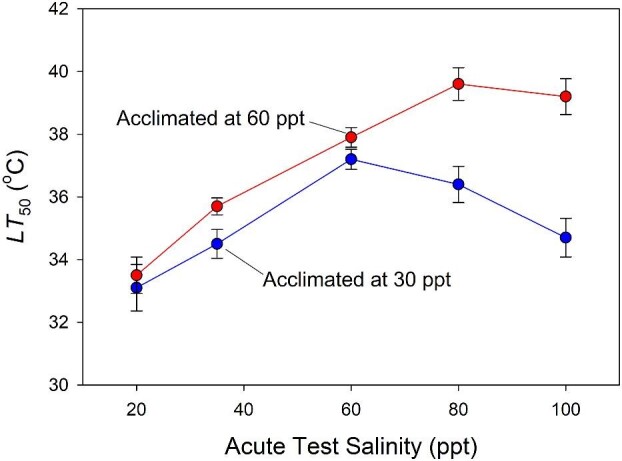
*LT*_50_ measured immediately after a shift to a new salinity varies with the salinity to which animals are acclimated. Blue symbols, animals acclimated to 30 ppt, red symbols, to 60 ppt. Error bars are standard errors.

Acute transfer of copepods to a range of test salinities had a dramatic effect on heat tolerance (Fig. 7). For individuals acclimated to 60 ppt, *LT*_50_ increased nearly monotonically with acute test salinity. The contrasting, dome-shaped relationship for copepods acclimated to 30 ppt resulted in a >4°C difference in *LT*_50_ at the highest acute test salinity (100 ppt) relative to individuals acclimated to 60 ppt.

### Carryover effects of a sublethal heat stress

Exposure of laboratory-maintained copepods to a 35°C thermal event significantly increased *LT*_50_ measured 1–3 days later (*P* < 0.01 in each case). The differences in mean *LT*_50_ between pre-stressed and unstressed animals was relatively small compared to the salinity effects described above (mean [s.d.]): 0.66 [0.30]°C, 0.56 [0.35]°C, and 0.69 [0.49]°C, for lags of 1, 2, and 3 days, respectively; the three means were not significantly different (1-way ANOVA, *P* = 0.834).

## Discussion

*T. californicus*’s humble splash-pool habitat harbors an intensely variable microenvironment where a variety of factors fluctuate dramatically—and unpredictably—within and between days. Our long-term field dataset reveals that *T. californicus*’s thermal physiology appears to keep pace with these dynamic changes, although salinity is a much better predictor of heat tolerance than is recent body temperature. Laboratory studies confirm the rapid and substantial plasticity of thermal tolerance in the face of realistic environmental shifts in salinity or temperature. However, further work is needed to adequately characterize the physical environment in other pools across *T. californicus*’s range, to confirm the biochemical mechanisms underlying plasticity, and to unravel more subtle fitness consequences of transient salinity and temperature fluctuations.

### Thermal tolerance of *T. californicus* keeps pace with rapid environmental fluctuations

Long-term environmental monitoring data reveal the variation faced by *T. californicus* in this particular splash-pool habitat. Environmental challenges can reach extraordinary levels, albeit in an unpredictable fashion. In our experimental pool, water/body temperature varies by as much as 21°C within a day (or as little as 1°C); daily maximum temperature can vary by as much as +10.5°C to −11°C from one day to the next. Similarly, pool salinity typically hovers near ambient seawater levels (mode ∼37 ppt), but it can change instantaneously by 29 ppt or by up to 49 ppt in one day. Although there is an overall seasonal trend in pool temperature, shifts in temperature, salinity, and the magnitude of one relative to the other can be predicted only 2–3 days in advance. The temperature and salinity variations imposed on *T. californicus* by its supratidal habitat far exceed those experienced by copepods in the oceanic mixed layer (Denny and Dowd 2022), and (for temperature) are similar to those experienced by sessile intertidal organisms such as mussels and limpets (e.g., Helmuth et al. 2002; Harley et al. 2009; Miller and Dowd 2017).

In the absence of similarly detailed temperature and salinity measurements for other pools, we cannot quantitatively assess how representative our experimental pool is of *Tigriopus* habitat in general. Given its small volume, shallow depth, and position high on the peak of an isolated outcrop, it is reasonable to suppose that our pool's environment is among the more variable and extreme of those along the shore at HMS. However, it is unlikely that its environment is atypical of those encountered by *Tigriopus* across its range. Central-California shores are renowned for their foggy summers, so our pool (at a relatively low latitude) may experience lower peak temperatures than those at higher latitudes that are routinely exposed to clear skies; e.g., Kelly et al. (2012) measured a temperature of 42°C at a pool in northern California. Furthermore, we have evidence that—for adult males from an even more northerly splash-pool population in Washington State—elevated salinity similarly confers elevated heat tolerance (W. Dowd and C. Terry, unpub. obs.). Documenting how multiple environmental stressors co-vary in pools across *Tigropus*’s range is, in our opinion, a necessary step toward both testing the generality of our results and exploring how environmental variation interacts with genetics to influence physiological evolution.

Our combined field and lab results suggest that (at least in our experimental pool) *T. californicus*’ physiology is adapted to the extraordinary pace and amplitude of environmental variation it encounters—these copepods exhibit rapid and reversible plasticity of heat tolerance over very short time scales. Notably, while both salinity and temperature shifts can prime copepods to tolerate subsequent thermal challenges, the effect of salinity dwarfs that of temperature. Our measurements of *LT*_50_ and knockdown temperature for freshly field-collected animals clearly demonstrate that thermal tolerance increases with increasing salinity (although knockdown temperature plateaus at above-average salinities). There are few available datasets that have repeatedly quantified tolerance limits of animals in naturally fluctuating conditions (Dowd and Denny 2020); most examples include only monthly or even seasonal sampling points (e.g., Bourget 1983; Udaka and Sinclair 2014). Field-based temporal series such as ours provide much needed context for single-factor, laboratory studies.

Driven predominantly by variation in salinity, the variation over short periods in thermal tolerance of a single splash pool's *Tigriopus* population is remarkably large: 7.5°C in the field, 6.4°C in the laboratory salinity study. It exceeds by a factor of two the level of variation in heat tolerance found at constant, benign salinity across *Tigriopus* populations collected from sites spanning 17° of latitude and reared in common garden conditions (∼3.5°C range of *LT*_50_ from central Oregon to central Baja populations; Kelly et al. 2012). Compared to previous studies, we also found a greater upper tolerance limit among Monterey Bay *Tigriopus* at typical seawater salinity (>37°C compared to no higher than 36.5°C in other west coast populations; Kelly et al. 2012, 2016). However, differences in protocols between studies necessitate caution when directly comparing these results (reviewed in, for example, Chung and Schulte 2020).

More broadly, the magnitude of salinity-induced plasticity in upper thermal limits we observed in *Tigriopus* is greater than is typically observed when organisms are acclimated in the laboratory to widely divergent temperatures (reviewed in Gunderson et al. 2016). The median temperature-induced plasticity of thermal limits (quantified as the temperature when righting reflexes are lost, rather than the lethal temperature) in that meta-analysis was 2.3°C. Temperature-induced plasticity exceeded 7.4°C in <5% of studies, and only when acclimation temperatures differed by at least 20°C.

The rapid response of thermal tolerance to environmental changes in *Tigriopus* is reminiscent of patterns observed in *Drosophila* (Overgaard and Sørensen 2008), where recent work highlights the potential for even single events to dramatically, and continuously, alter population genetic structure via selection (Bergland et al. 2014; Rudman et al. 2022). In light of its short generation time (23–26 days [Edmands and Harrison 2003]), *Tigriopus* is sometimes compared to *Drosophila*. Could rapid evolution account for the fluctuations in thermal tolerance we observe in the field? Several lines of evidence suggest that this is unlikely. First, our two laboratory studies clearly show that such shifts of thermal tolerance can arise rapidly in the absence of mortality/selection. Second, at any given salinity, we consistently found a narrow range of lethal temperatures among freshly collected animals (Fig. 6); in our pool there appears to be relatively little phenotypic variation among individuals from which to select (although there may be greater variation within the metapopulation to which our pool's population is connected). This conclusion is bolstered by the fact that *Tigriopus* appear to have a limited capacity to evolve higher thermal tolerances. Artificial selection studies in *Tigriopus* have revealed minimal capacity for evolution of enhanced thermal tolerance (on the order of 0.1–0.5°C; Kelly et al. 2012, 2013, 2016, 2017), at least in part due to limited standing genetic variation within each local population (Edmands and Harrison 2003; Kelly et al. 2013). The lack of evidence for robust responses to artificial selection over several generations renders rapid evolution an unlikely explanation for the large adjustments in thermal tolerance we observed over short intervals in the field. Thus, we tentatively conclude that physiological plasticity is the most parsimonious explanation for our field observations.

### Rapid responses of thermal tolerance to salinity and temperature shifts

Our field monitoring results suggest that *T. californicus* can take advantage of the interaction among salinity, sublethal thermal stress, and thermal tolerance, triggering its physiology to keep pace with the rapid, unpredictable fluctuations of its hypervariable physical environment. In particular, these animals may capitalize on the cross correlation between salinity and pool temperature. Although neither temperature nor salinity is reliably predictable more than 2–3 days in advance, when temperature is high there is a substantial likelihood that salinity is also high (Fig. 4B). Our field sampling dates were separated by at least 2 days (median 9 days), preventing accurate quantification of the pace of plastic changes in nature. However, we filled this temporal gap with complementary laboratory studies, which confirmed that the responses of *Tigriopus*' thermal physiology to salinity and/or temperature change are swift. Adjustments in thermal tolerance are completed in less than 24 h following a sublethal temperature exposure and perhaps in as little as 1–2 h upon acute salinity transfer.

Interactive effects of salinity on heat tolerance have been demonstrated in a variety of marine invertebrates including anemones (Gegner et al. 2017), crabs (Todd and Dehnel 1960; Tagatz 1969), and estuarine copepods (Bradley 1978); in bacteria (e.g., Diamant et al. 2001); and even extending as far as agricultural crops such as wheat (e.g., Song et al. 2005). By contrast, salinity acclimation had no effect on the upper critical temperature for heart-rate failure in intertidal *Mytilus* mussels (Braby and Somero 2006), and salinity may have less impact on the heat tolerance of bony fishes that effectively osmoregulate (e.g., Davis et al. 2019; Hines et al. 2019). In several instances of cross-tolerance between these two factors there is evidence of the underlying mechanism(s), often involving accumulation of compatible solutes and/or induction of the molecular-chaperone response (e.g., Diamant et al. 2001).

The precise mechanism for a rapid, salinity-induced effect on heat tolerance in *Tigriopus* remains hypothetical, but there is substantial circumstantial evidence that accumulation of small organic solutes (i.e., osmolytes) including amino acids plays a role. Like most marine invertebrates, *Tigriopus* is an osmoconformer; as the salinity of the surrounding water increases, these copepods increase the osmolality of their intracellular milieu by accumulating small amino acids, primarily proline and alanine (Burton and Feldman 1982, 1983; Burton 1991; Willett and Burton 2002; DeBiasse et al. 2018; Lee et al. 2020). The adjustment is measurable within 10 min and essentially complete in 3–6 h for salinities ranging from 6–80 ppt. Proline has been shown to act as a thermoprotectant, shielding proteins (including enzymes) from denaturation (Somero et al. 2017). Thus, it is likely that the proximal response to salinity—a rapid increase in proline concentration—has the indirect benefit of increased thermal tolerance. The exact manner in which proline might affect thermal tolerance (which specific proteins it affects and how they contribute to tolerance) is complex and not completely understood (Willett and Burton 2002; DeBiasse et al. 2018; Lee et al. 2020). Importantly, these osmolyte studies have not yet been replicated at the extreme hypersaline range of conditions where thermal tolerance is so dramatically elevated in our population. Our data also suggest there might be limits to these compensatory processes that roughly mirror the greatest magnitudes of salinity change encountered in the splash pool. Specifically, we observed that very large, artificially imposed changes in salinity beyond the bounds of observed day-to-day environmental shifts did not always have the expected effect on heat tolerance; for example, see the nearly flat response of *LT_50_* for acute transfer from 30 ppt to 100 ppt in Fig. 7.

While osmolytes clearly warrant further study in this context, there may be other mechanisms contributing to the enhancement of *Tigriopus'* elevated heat tolerance under high salinity. For example, Kelly et al. (2017) provide evidence for rapid elevation of transcript levels for several heat shock proteins following a 1-h exposure to elevated salinity. Thus, a combination of osmolyte and molecular-chaperone effects could explain the elevation of heat tolerance in *Tigriopus* in hypersaline conditions.

The mechanisms through which salinity interacts with knockdown temperature are less obvious, particularly given that the relationship is flat at salinities above 40 ppt. The observation that copepods rapidly recover even from 5 repeated knockdown episodes (within 1–2 min) suggests that the acute knockdown has a neurological basis (as in *Drosophila* [Jørgensen et al. 2020] and zebrafish [Andreassen et al. 2020]), rather than causing irreversible macromolecular damage.

In addition to using salinity as a signal by which to prepare for elevated temperatures, exposure to an elevated (but sublethal) temperature can also induce an increase in thermal tolerance over environmentally relevant time scales. Kelly et al. (2017) reported that exposure to a 34°C heat shock elicited an increase in *LT*_50_ a day later. Our experiments (with a 35°C initial shock) confirm and expand upon this result. The carryover effect persists for at least 3 days, sufficient to act as a potential prophylactic response for the majority of intervals between the imposition of stressful temperatures (Fig. 3B). Carryover effects with similar, environmentally pertinent temporal scales have been noted for intertidal mussels (Moyen et al. 2020). Several questions remain, however, about the efficacy of this response. First, in nature, a daily maximum temperature of 34–35°C is sufficiently rare that it is likely followed by substantially lower temperatures in the following days, temperatures requiring less thermal tolerance. The elevated tolerance elicited by a heat shock of 34–35°C is, therefore, of questionable benefit (see the discussion of safety margins below). Second, although temperatures of 34–35°C are sublethal, they far exceed “typical” temperatures in this splash pool environment, and the effect of lower, more often encountered, temperatures is unclear. Studies of developmental plasticity showed a small effect of repeated exposure to 28°C for 6 h each day throughout development; *LT_50_* increased, but by <1°C for several *Tigriopus* source populations (Kelly et al. 2012). The lack of any discernible influence of daily maximum temperature on the pattern of *LT*_50_s recorded from our field-fresh copepods suggests that run-of-the-mill temperatures (e.g., the modal daily maximum of 26°C in our pool) do not substantially affect *LT*_50_. Third, it remains to be seen how long the beneficial effect of a single sublethal temperature might persist, given that a nontrivial fraction of extreme events (∼30% of events ≥30°C, Fig. 3B) are separated by intervals longer than 3 days. Lastly, these lab-measured carryover effects were evaluated at only a single test salinity; it is possible that the magnitude and/or duration of the effect varies with salinity.

### The conundrum of environmentally induced expansion of the thermal safety margin

The strong effect of ambient salinity, and to a lesser extent temperature, on thermal tolerance poses a conundrum: Why would copepods *need* to enhance their thermal tolerance when “baseline” thermal tolerance (tolerance at average salinity) is already sufficient? Indeed, at all but the lowest salinities (below 20 ppt) median lethal temperature (*LT*_50_) exceeds the maximum measured pool temperature as well as the estimated theoretical maximum temperature of 36.4°C. The reasons for this thermal “safety margin” are elusive, but similar or larger safety margins have been observed in intertidal limpets (Miller et al. 2009; Denny and Dowd 2012), mussels (Denny et al. 2011), and low-intertidal crabs (Stillman and Somero 2000) (although the margins in high intertidal crabs are smaller [Stillman and Somero 2000]). Theory predicts a cost to maintaining the capability of surviving greatly elevated temperatures (e.g., fitness tradeoffs at lower temperatures; Willett 2010); yet, this physiological safety margin persists.

It is possible that rare selective events could account for this persistence. We previously simulated long-term environmental patterns and costs of maintaining and inducing thermal defenses in an intertidal limpet to illustrate that even extremely rare high-temperature events are capable of selecting for a significant thermal safety margin (Denny and Dowd 2012). There are undoubtedly splash pools that potentially have more extreme thermal conditions than those in our experimental pool. Indeed, as noted above, Kelly et al. (2012) recorded temperatures up to 42°C in splash pools in Northern California, slightly above the maximum *LT*_50_ measured in our population at high salinity, and well above the *LT*_50_ at average salinity. Rare extreme events in the most stressful local pools could select for a genotype that is highly heat-tolerant. Local metapopulations continuously replenish populations within individual, ephemeral pools (Dybdahl 1994; Burton 1998), potentially delivering such genotypes to other more benign pools. In this manner, selection by rare events in the local metapopulation could account for the baseline safety margin. If this is the case, the drastically enhanced thermal tolerance associated with high salinity likely arises not as a result of selection for high thermal tolerance *per se*, but rather as a byproduct of physiological osmoregulatory mechanisms activated when coping with high salinity (see above). To test these ideas, it will be necessary to quantify the variation in extreme temperatures among pools of known gene flow.

Despite their obvious interest in the context of climate change, thermal safety margins for survival (as assessed by *LT*_50_) may be of limited biological relevance. Because it notes only whether an organism is alive or dead, *LT*_50_ is a blunt instrument—it almost certainly fails to capture important aspects of thermal performance. Instead, consequences associated with elevated “thermal tolerance” may well play out in other aspects of fitness, by impacting processes such as growth and reproduction. For example, Egloff (1966) found that for salinity above average (50 ppt, at which *LT*_50_ in our pool is elevated to ∼38°C), egg production in *T. californicus* was reduced except at low temperature (15°C). While this result suggests that increased thermal tolerance induced by high salinity may have negative impacts at benign temperatures, it must be interpreted with caution, because it was derived from unrealistically constant laboratory exposures; in nature, extraordinary values of salinity and temperature are experienced only transiently. Notably, Kelly et al. (2013) illustrated that selection for increased thermal tolerance by acute high-temperature events tended to enhance, rather than decrease, some fitness-related performance traits (body size, clutch size, and starvation resistance), although the response to selection was small. By contrast, Seigle et al. (2022) show that repeated exposure to 32°C did not affect survivorship in *T. californicus* relative to exposure to 26°C, but animals exposed to the higher (but still sublethal) temperature produced fewer offspring. Further information from more realistic experiments is required to better understand the fitness costs and benefits of salinity-influenced thermal tolerance.

Of course, to fully understand environmental influences on the physiology and performance of *Tigriopus* we must ultimately consider the other abiotic (e.g., oxygen concentration and pH) and biotic factors (e.g., quantity and composition of the microalgal diet) that fluctuate in their microhabitat (Kroeker et al. 2017; Kroeker and Sanford 2022). Both pH and oxygen concentration vary through the day, being lower at night as photosynthesis by pool algae ceases and respiration by the community continues. In the middle of the day, when temperatures reach their maximum, the *Tigriopus* population in our experimental pool typically experiences an alkaline, hyperoxic environment (mean pH = 8.56; mean oxygen concentration = 20.9 mg/L, approximately 215% saturation). Prior work suggests that dissolved oxygen concentration plays little role in upper thermal tolerances under environmentally relevant conditions similar to those measured here (Dinh et al. 2020), although those observations were limited to a single salinity and pH. These observations have only scratched the surface. The effects of simultaneous variation in temperature, salinity, oxygen concentration, pH, and community interactions remain to be more thoroughly determined.

## Conclusion

Long-term, temporally detailed measurements of both the physical environment and the corresponding physiological capabilities of organisms are key not only to elucidating the mechanisms that allow these animals to function in variable environments, but also to identifying possible constraints on function. The copepod *T. californicus* adjusts rapidly to salinity and temperature shifts in its extraordinarily dynamic environment. Importantly, the remarkable degree of thermal-tolerance plasticity we observed would be overlooked if one manipulated only temperature, rather than opportunistically sampling across the natural range of salinity. These data complement the wealth of information already available on the mechanisms of osmoregulation and the latitudinal patterns of salinity and temperature tolerance in this species, yet they call for a more nuanced and environmentally grounded approach to climate-change biology than the study of any one static environmental factor in isolation. Furthermore, if our ultimate goal extends beyond this single species to a mechanistic forecast of how the entire splash-pool community will respond to change, much work remains to fully integrate our burgeoning understanding of *T. californicus*’s physiology with corresponding information regarding the other species it interacts with in its habitat.

## Supplementary Material

obac037_Supplemental_File

## Data Availability

Data are available on Mendeley Data: doi: 10.17632/p7d5b4vbrh.1.
