# Supplementary material for: Elevated Salinity Rapidly Confers Cross-Tolerance to High Temperature in a Splash-Pool Copepod
Source: Integr Org Biol. 2022 Aug 6;4(1):obac037. doi: 10.1093/iob/obac037 (PMC9394168; doi:10.1093/iob/obac037)
Supplement: obac037_Supplemental_File [file obac037_supplemental_file.docx]

**Supplement**

**Pool Surface Area**

The topography of our experimental pool was such that its surface area increases in approximately linear proportion to its volume (Fig. S1). Given the wide variety of pool topographies in which *T. californicus* occurs, this relationship is unlikely to be generally true.

**Additional Environmental Data**

***Salinity***

The salinity of our experimental pool ranged from 8 ppt to 190 ppt in a pattern that varied both seasonally and from day to day (Fig. S2).

***Pool Temperature***

The distribution of daily maximum temperatures is distinctly bimodal, with modes of 16 and 26^o^C (Fig. S3A). The rate of temperature ranged from 2 to 10 ^o^C hr^-1^, on average increasing with increasing daily maximum temperature (Fig. S3B). The rate of cooling ranged from -3 to -15^o^C hr^-1^, and was negatively correlated with daily maximum temperature (Fig. S3B). The overall trend of maximum daily temperatures had a distinct seasonal pattern, reaching a peak in late summer and early autumn and a nadir in midwinter (Fig. S3C). Daily minimum temperatures followed a seasonal pattern similar to that of maximum temperature (Fig. S3C), although the peak of the overall trend was shifted to later in the year. The average daily range of temperatures was 9.3^o^C, with a maximum of 21^o^C and a minimum of 1^o^C. Temperature range had a seasonal trend similar to that of daily maximum temperature (Fig. S3D), but there was considerable day-to-day variation around that trend. For example, despite the occurrence of the largest temperature ranges in summer, these drastic thermal excursions were interspersed with days in which temperatures varied little. The overall distribution of pool temperatures is shown in Fig. S4A. The distribution of daily minimum temperatures ranged from 6.5 to 19.5^o^C with modes at 10 and 16^o^C (Fig. S4B).

**Additional Time Series Analyses**

***Methods***

*Conditional Probability of Stress:* In addition to temporal correlations (ACF, CCF), we also calculated the conditional probability that, if a given maximum temperature was encountered on a given day, a potentially stressful temperature would be encountered 1, 2, or 3 days later, or any time in that 3-day window. We assume that temperatures are potentially stressful if they are extreme for this particular environment. What is “extreme” is open to interpretation (Dowd and Denny 2020). We use two values: 24.5^o^C is a temperature commonly found to elicit increased production of heat-shock proteins in a variety of intertidal animals (e.g., snails, Tomanek and Somero 1999; mussels, Buckley et al. 2001; barnacles, Berger and Emlet 2007; limpets, Dong et al. 2008), and is the temperature delineating the top 5% of all temperatures measured (corresponding to the top 30% of daily maxima). For comparison we use an even more extreme temperature threshold (30^o^C), the top 0.5% of all recorded temperatures (corresponding to the top 5% of daily maxima). To estimate conditional probabilities, for each daily maximum temperature in the time series we noted (1) if there was a recorded daily maximum temperature 1, 2, or 3 days in the future and (2) whether the maximum temperature 1, 2, or 3 days in the future was ≥ 24.5^o^ or 30^o^C. The number of instances in which temperature exceeded the threshold 1, 2, or 3 days in the future (or on any of those days), divided by the number of days in which a maximum temperature was recorded, is an estimate of the conditional probability of such an event. The 95% confidence limits of these estimates were calculated according to Eqs. 24.28 and 24.29 of Zar (1999).

*Statistics of Extremes*: Our environmental measurements extended over only 2.5 years. However, the statistics of extremes allows one to extrapolate from measured data to predict the likelihood that more extreme conditions will be encountered by chance over longer intervals (Coles 2001). We used the *R* package *in2extRemes* and a points-over-threshold (POT) approach to estimate the shape ($\varepsilon$) and scale (*σ*) parameters that best fit the generalized Pareto distribution to our daily maximum temperature data. If $\varepsilon<0$, the distribution’s upper tail is bounded, indicating that (given current variability) there is an absolute maximum value, *T*_max,abs_, that will not be exceeded no matter how long one waits:

$T_{max,abs}=\mu-\frac{\sigma}{\varepsilon}$, $(\varepsilon<0)$ Eqn. S1.

where $\mu$ is the threshold temperature used to define what is “extreme” in the estimation. Based on data diagnostics, we used a threshold of 30^o^C (delimiting the top 5% of daily maxima), although the results were insensitive to the precise choice of threshold. The data were declustered prior to analysis to avoid double counting single events. Confidence intervals were estimated using 500 iterations of a parametric bootstrap.

***Results***

*Conditional Probability of Stress:* The occurrence of a thermal event above 24.5^o^C within the next 3 days is reasonably predictable (*p* > 0.5) only if the current day's maximum temperature exceeds approximately 24^o^C (Figure S5), providing little forewarning of increasingly stressful conditions. Similarly, occurrence of daily maxima ≥ 30^o^C within the next three days is reasonably predictable only if the current day's maximum exceeds 27^o^C, and the level of predictability is lower.

*Statistics of Extremes*: The statistics of extremes suggests that—given current environmental variability—it is unlikely that copepods in our experimental pool will encounter temperatures substantially in excess of those encountered in our short time series. The maximum temperature estimated to occur in 100 events ≥ 30^o^C is 35.71^o^C (95% CL: 34.32, 37.30^o^C), essentially the same as our measured maximum temperature (36$\pm$0.5^o^C). In the 624 days of our measurements, 32 had temperatures $\geq$ 30^o^C, a rate of 0.051 day^-1^. At this rate, on average it would take 5.34 years for the pool to accumulate 100 such events, the equivalent of 70 – 90 copepod generations. The best-fit estimate for the shape parameter $\varepsilon$ is -0.585, significantly < 0, (95% CL: -1.179, -0.263), suggesting that, given current environmental variability, there is an absolute maximum temperature for our pool. The best-fit estimate of the scale parameters, σ, is 3.718 (95% CL: 2.234, 6.579). The estimated absolute maximum temperature is, thus, 36.4^o^C (Eqn. 1), again essentially the same as the maximum temperature we measured.

*Future Extremes.* Calculation of thermal safe margins requires an estimate of the extreme temperatures an organism is likely to encounter. Our estimate of the absolute maximum temperature that could by chance occur in our pool (36.4^o^C) assumes that the pattern of environmental variation that governs pool temperature remains statistically stationary into the future (Coles 2001). It is tempting to suppose that the increasing air temperatures typical of climate change would elevate this value, decreasing the safety margin. This may indeed be the case. However, air temperature is just one of several variables controlling pool temperature, and it is unclear how co-occurring changes in wind speed, cloud cover, sea-surface temperature, sea-level, relative humidity, and wave height will combine to affect future extreme values. As with physiology, a deep, mechanistic understanding of splash-pool thermal mechanics will be required to accurately predict the consequences of climate change for *T. californicus*.

**Rates of Change of Salinity**

***Theory***

Splash-pool copepods’ physiology is potentially affected by the rate at which salinity changes due to evaporation. Three simple scenarios provide insight into how the rate of change of salinity depends on pool topography. Each scenario begins with the definition of salinity, *S*:

$S=\frac{m}{M+m}$ (Eqn. S2)

where *m* is the mass of salt and *M* the mass of water in the pool. As water evaporates, salt remains, so *m* is constant. Thus, the rate of salinity change depends solely on the rate of evaporative water loss. For simplicity, we assume that the rate of evaporation (*dM/dt)* is a linear function of pool surface area, *A*:

$\frac{dM}{dt}=-EA$. (Eqn. S3)

The evaporation coefficient *E* (mass per area per time) depends on wind speed, pool temperature, and relative humidity, all of which we assume are constant. At high salinities, evaporation will also be a function of salinity itself, but for simplicity we ignore this factor.

*Constant Shape*: If the pool’s topography is such that pool shape is independent of pool volume (e.g., it is conical), its surface area is proportional to *M*^2/3^ such that $A=k_{1}M^{2/3}$. Thus,

$$\frac{dM}{dt}=-E{k_{1}M}^{2/3}$$

$$\int M^{-2/3}dM=-Ek_{1}\int dt$$

$$3M^{1/3}=-Ek_{1}t+3{M_{0}}^{1/3}$$

$$M\left( t \right)=\left( {M_{0}}^{1/3}-\frac{Ek_{1}t}{3} \right)^{3}$$

$S\left( t \right)=\frac{m}{\left( {M_{0}}^{1/3}-\frac{Ek_{1}t}{3} \right)^{3}+m}$ (Eqn. S4)

Here *M*_0_ is initial pool volume. In this scenario, the ratio of surface area to water mass is $k_{1}M^{-1/3}$, which increases rapidly as mass evaporates; thus the rate of salinity increase itself increases rapidly through time. As $\frac{Ek_{1}t}{3}$ approaches ${M_{0}}^{1/3}$, this equation becomes increasingly inaccurate because it ignores the effect of salinity on the rate of evaporation.

*Constant Area*: If the pool’s topography is such that pool’s surface area is independent of volume (e.g., it is cylindrical):

$$\frac{dM}{dt}=-EA$$

$$\int dM=-EA\int dt$$

$$M\left( t \right)=-EAt+M_{0}$$

$S\left( t \right)=\frac{m}{M_{0}-EAt+m}$ (Eqn. S5)

In this scenario, salinity increases approximately hyperbolically with time. As *EAt* approaches $M_{0}$, this equation becomes increasingly inaccurate, again because it ignores the effect of salinity on the rate of evaporation.

*Area Proportional to Volume*: If surface area is directly proportional to pool volume (as it is in our experimental pool, Fig. S1), *A* = *k_2_M*:

$$\frac{dM}{dt}=-Ek_{2}M$$

$$\int\frac{1}{M}dM=-Ek_{2}\int dt$$

$$\ln M=-Ek_{2}t+\ln M_{0}$$

$$M\left( t \right)=M_{0}e^{-Ek_{2}t}$$

$S\left( t \right)=\frac{m}{M_{0}e^{-Ek_{2}t}+m}$ (Eqn. S6)

If *m* is small compared to *M*(*t*),

$$S\cong\frac{m}{M}$$

$S\left( t \right)\cong S_{0}e^{Ek_{2}t}$, (Eqn. S7)

where *S*_0_ is initial pool salinity. Taking the logarithm of each side of Eqn. S7, we see that

$\ln S\left( t \right)\cong Ek_{2}t\ln S_{0}$ (Eqn. S8)

Thus, if pool surface area is proportional to pool volume, plotting the log of salinity as a function of time should yield a straight line. This is indeed true for our experimental pool (Fig. S6).

The approximation given in Eqn. S7 overestimates salinity as a function of time, but at the salinities typically encountered in splash pools the error ($\Delta S=$Eqn. S7 - Eqn. S6) is slight: $\Delta S=$0.8 ppt at a salinity of 50 ppt, 3 ppt at 75 ppt, and 7 ppt at 100 ppt. At higher salinities, the error becomes quite substantial. Whereas Eqn. S6 asymptotes to 1000 ppt (i.e., there is no water left), Eqn. S7 mistakenly trends toward infinity.

**Supplement Figure Legends**

**Figure S1. In our experimental splash pool, surface area increases approximately linearly with pool volume.** Area (m^2^) = 0.0297 x Volume (L) (*r*^2^ = 0.973).

**Figure 2. Salinity varies through time.** A. The overall distribution of salinities. B. Elevated salinities occurred primarily in April–October across the 2.5 years of monitoring data. C. Time series of salinities; September 2021 through January 2022. Note the interventions in which seawater was added during an episode of unusually calm seas.

**Figure S3. Pool temperature varies through time.** A. The distribution of daily maximum temperatures is bimodal, with one mode approximately equal to ambient seawater temperature (16^o^C) and the other approximately equal to average daily maximum temperature (26^o^C). B. Increasing and decreasing rates of change are correlated with daily maximum temperature. On average, the rate of temperature increase is Δ*T* = 0.3727*T*_max_ - 4.9827, where *T*_max_ is daily maximum temperature (*r*^2^ = 0.321). On average, the rate of temperature decrease is Δ*T* = -0.5873*T*_max_ + 10.52 (*r*^2^ = 0.340). C. The pattern of daily maximum (red dots) and minimum (blue dots) temperatures through the year. Solid black lines are polynomial fits to the data: for maxima *T*_max_ = -4.277x10^-4^*YD*^2^ + 0.159*YD* + 12.246 (*r*^2^ = 0.711); for minima *T*_max_ = -1.068x10^-6^*YD*^3^ + 4.048x10^-4^*YD*^2^ - 0.017*YD* + 9.463 (*r*^2^ = 0.725). *YD* = year day. D. Daily range of pool temperature (*TR* = maximum – minimum) varies through the year in parallel with daily maximum temperature: *TR* = -2.388x10^-4^*YD* + 0.0830*YD* + 5.091 (*r*^2^ = 0.464).

**Figure S4. Temperature distributions.** A. The overall distribution of pool temperatures is unimodal with a mode of 12^o^C. B. The distribution of daily minimum temperatures is bimodal, with modes of 11 and 16^o^C.

**Figure S5. The conditional probability of occurrence rises with current-day maximum temperature.** Values are for temperatures ≥ 24.5^o^C (blue) or 30^o^C (red) A. Probability 1 day in the future. B. Probability 2 days in the future. C. Probability 3 days in the future. D. Probability of occurrence anytime within the next 3 days. Dashed lines are 95% confidence limits.

**Figure S6. In accordance with theory, the rate of salinity increase due to evaporation rises exponentially with time in our experimental pool.** Consequently, the natural logarithm of salinity rises linearly with time (*r*^2^ = 0.973).

**Supplement Figures**

**
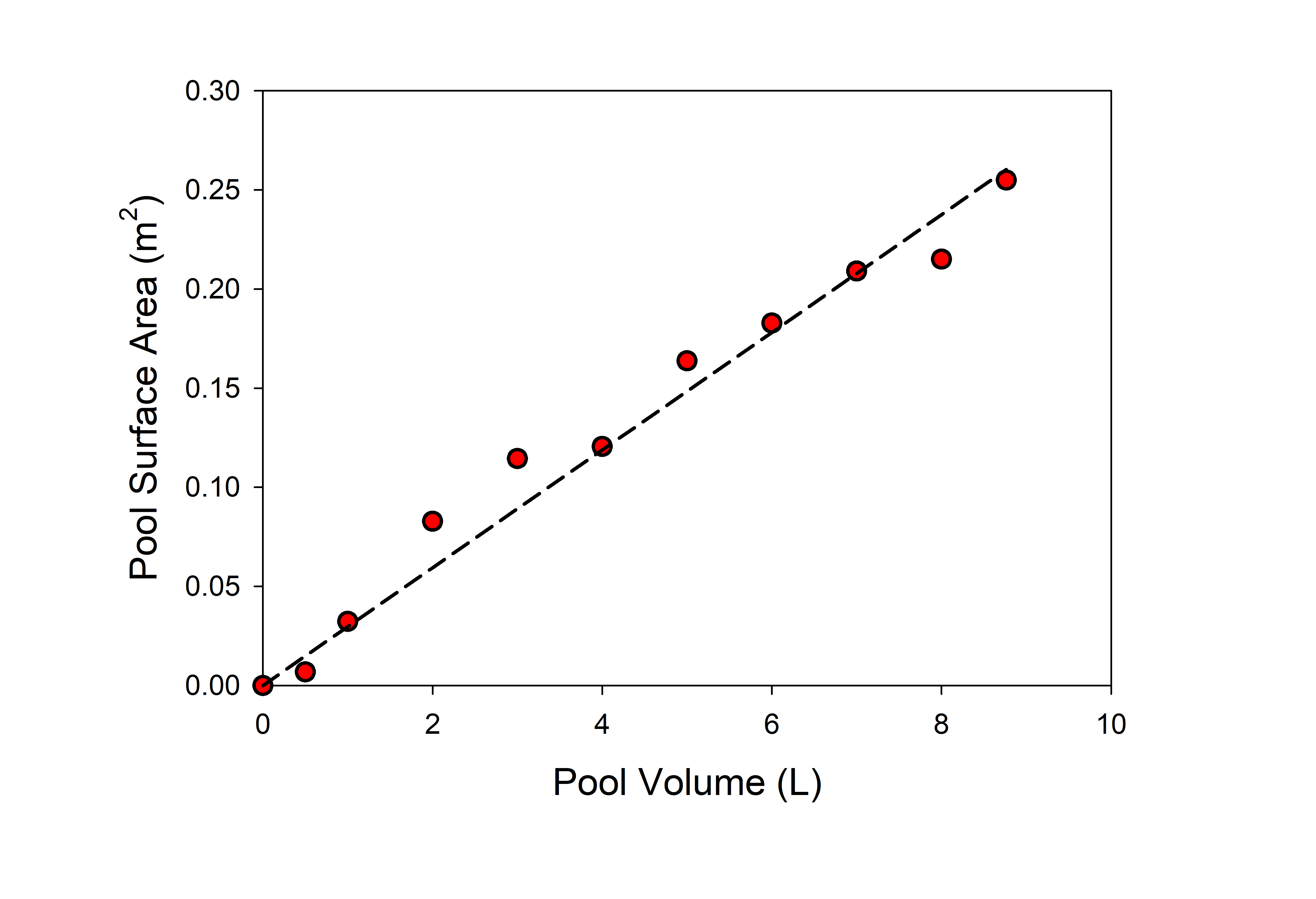
**

**Figure S1. In our experimental splash pool, surface area increases approximately linearly with pool volume.** Area (m^2^) = 0.0297 x Volume (L) (*r*^2^ = 0.973).

**
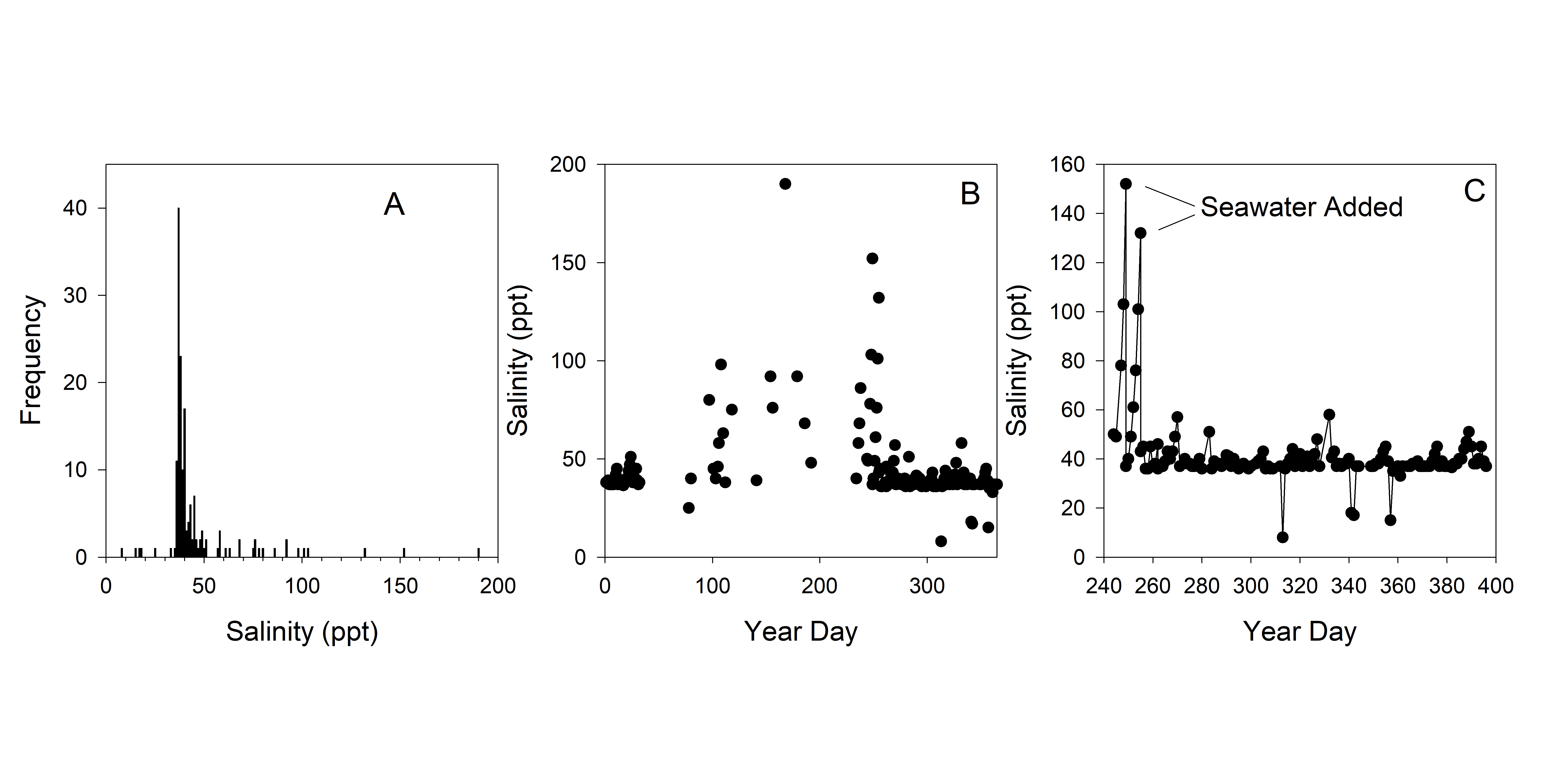
**

**Figure 2. Salinity varies through time.** A. The overall distribution of salinities. B. Elevated salinities occurred primarily in April–October across the 2.5 years of monitoring data. C. Time series of salinities; September 2021 through January 2022. Note the interventions in which seawater was added during an episode of unusually calm seas.


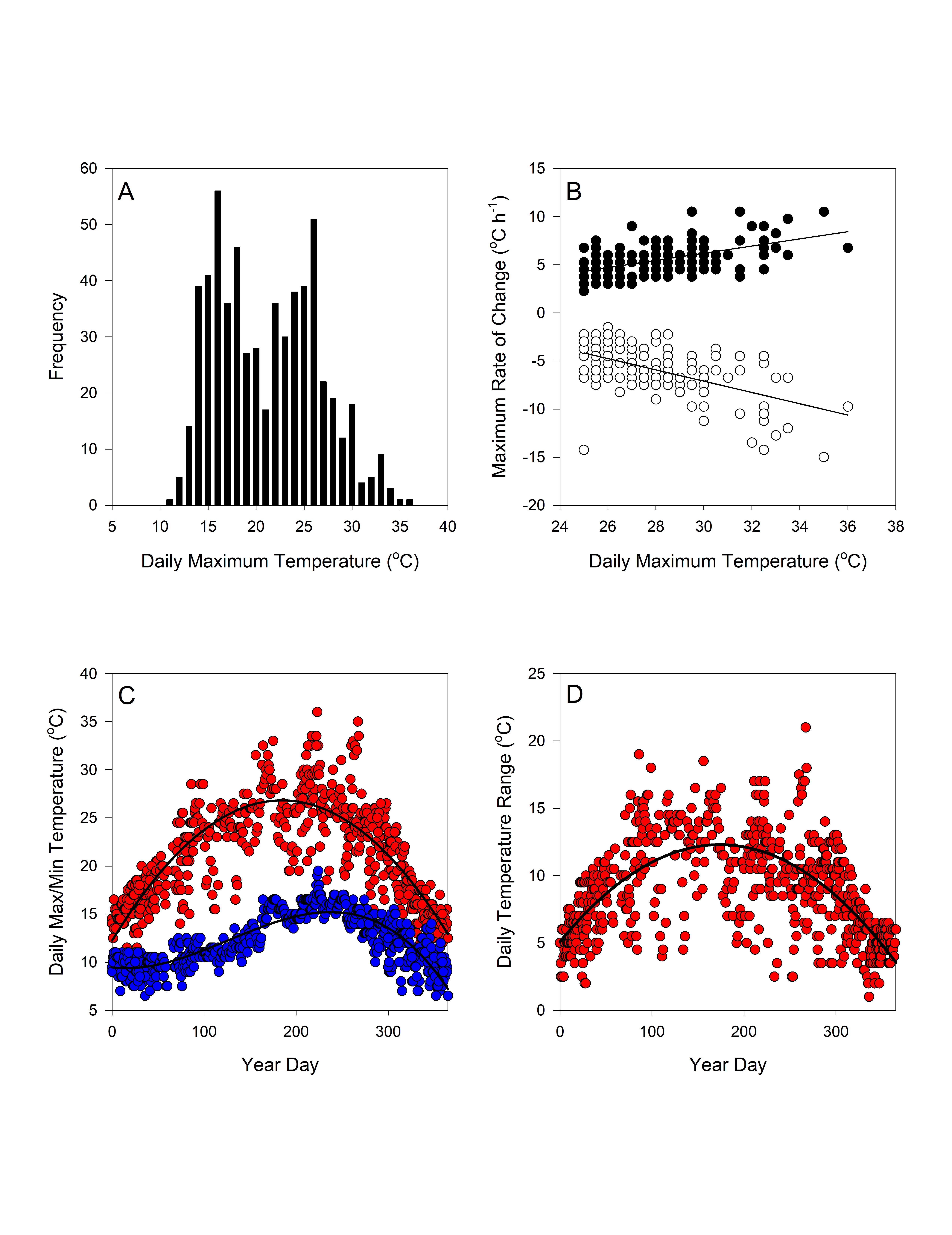


**Figure S3. Pool temperature varies through time.** A. The distribution of daily maximum temperatures is bimodal, with one mode approximately equal to ambient seawater temperature (16^o^C) and the other approximately equal to average daily maximum temperature (26^o^C). B. Increasing and decreasing rates of change are correlated with daily maximum temperature. On average, the rate of temperature increase is Δ*T* = 0.3727*T*_max_ - 4.9827, where *T*_max_ is daily maximum temperature (*r*^2^ = 0.321). On average, the rate of temperature decrease is Δ*T* = -0.5873*T*_max_ + 10.52 (*r*^2^ = 0.340). C. The pattern of daily maximum (red dots) and minimum (blue dots) temperatures through the year. Solid black lines are polynomial fits to the data: for maxima *T*_max_ = -4.277x10^-4^*YD*^2^ + 0.159*YD* + 12.246 (*r*^2^ = 0.711); for minima *T*_max_ = -1.068x10^-6^*YD*^3^ + 4.048x10^-4^*YD*^2^ - 0.017*YD* + 9.463 (*r*^2^ = 0.725). *YD* = year day. D. Daily range of pool temperature (*TR* = maximum – minimum) varies through the year in parallel with daily maximum temperature: *TR* = -2.388x10^-4^*YD* + 0.0830*YD* + 5.091 (*r*^2^ = 0.464).


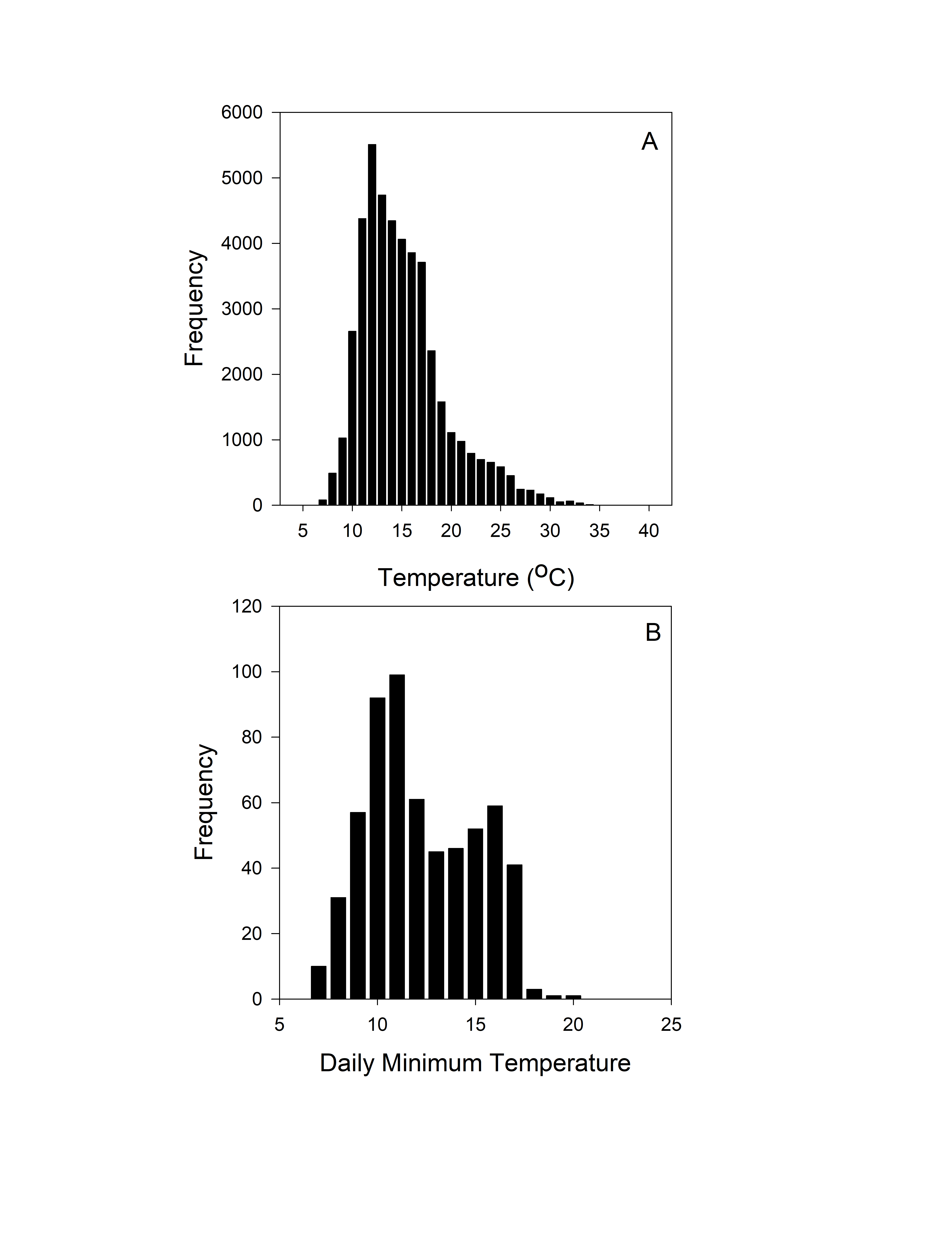


**Figure S4. Temperature distributions.** A. The overall distribution of pool temperatures is unimodal with a mode of 12^o^C. B. The distribution of daily minimum temperatures is bimodal, with modes of 11 and 16^o^C.


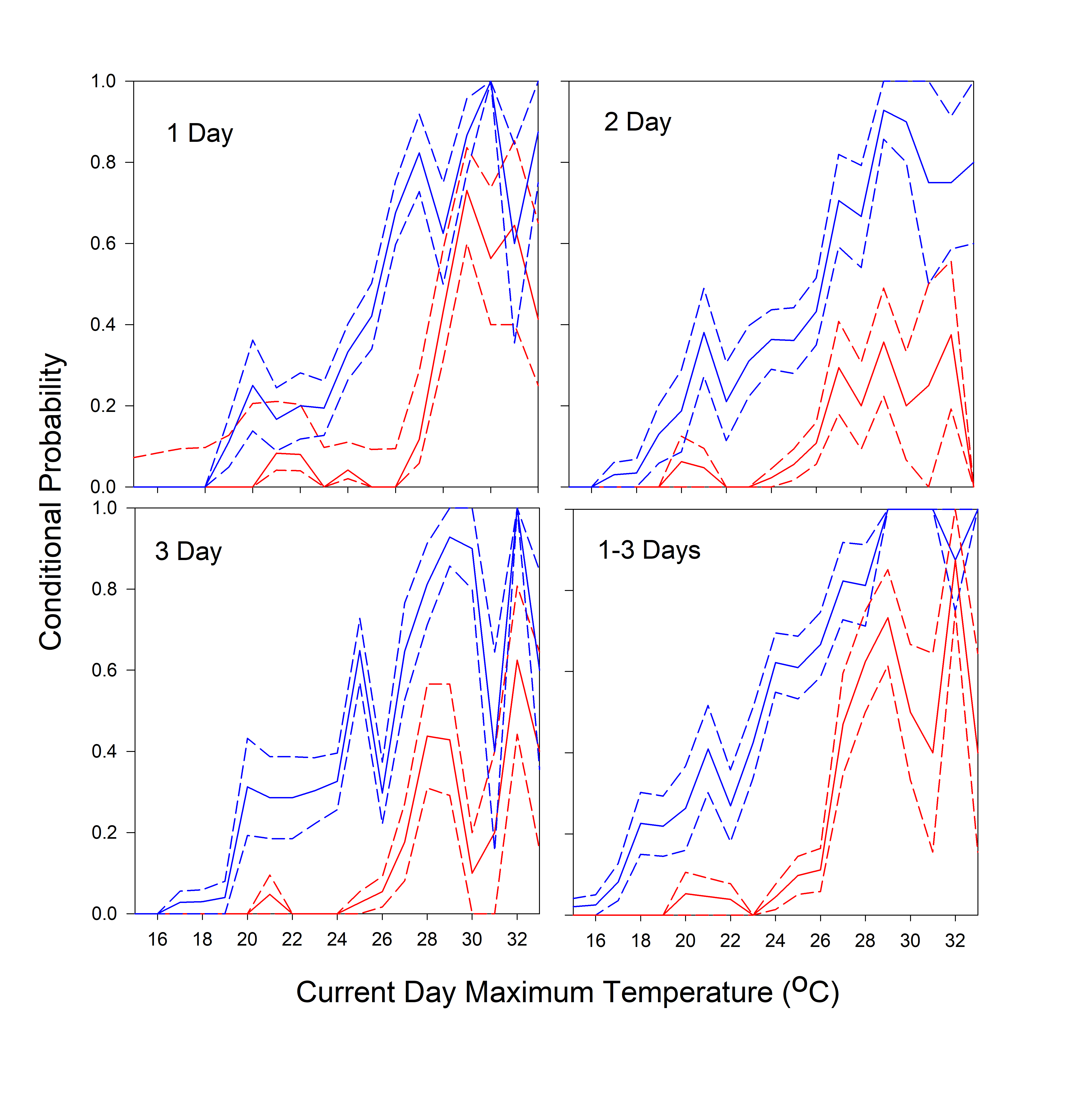


**Figure S5. The conditional probability of occurrence rises with current-day maximum temperature.** Values are for temperatures ≥ 24.5^o^C (blue) or 30^o^C (red) A. Probability 1 day in the future. B. Probability 2 days in the future. C. Probability 3 days in the future. D. Probability of occurrence anytime within the next 3 days. Dashed lines are 95% confidence limits.


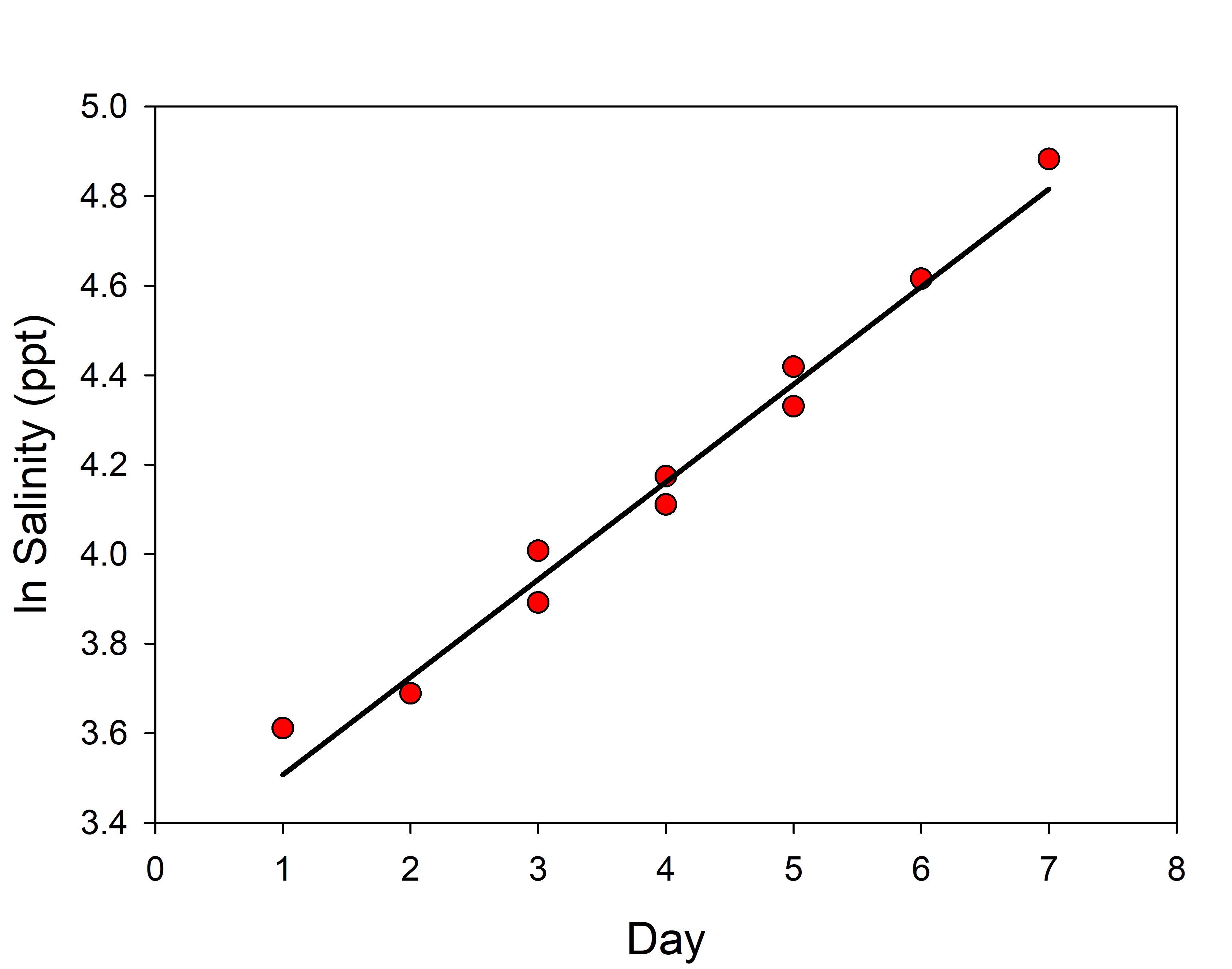


**Figure S6. In accordance with theory, the rate of salinity increase due to evaporation rises exponentially with time in our experimental pool.** Consequently, the natural logarithm of salinity rises linearly with time (*r*^2^ = 0.973).
